# Mitigating mitochondrial dysfunction: a novel strategy for Chinese botanical drugs against osteoporosis

**DOI:** 10.3389/fphar.2026.1809770

**Published:** 2026-05-14

**Authors:** Shiyu Li, Hongyu Liu

**Affiliations:** 1 Rehabilitation Department, Central Hospital of Dalian University of Technology, Dalian Municipal Central Hospital, Dalian, Liaoning, China; 2 Department of Pharmacy, Central Hospital of Guangdong Prison, Guangzhou, Guangdong, China

**Keywords:** mitochondrial dysfunction, osteoporosis, bone homeostasis, osteoblast, osteoclast, chinese botanical drugs

## Abstract

Osteoporosis (OP) is a chronic metabolic disease characterized by reduced bone mass and impaired bone microstructure, posing a significant global health threat. Mitochondria, as the body’s energy regulators, participate in numerous critical biological processes, and their dysfunction is a precipitating factor in various diseases. Accumulating evidence indicates that mitochondrial dysfunction—including abnormalities in mitochondrial biogenesis, dynamics, oxidative stress, and mitophagy—play a pivotal role in OP pathogenesis. Chinese botanical drugs (CBDs), leveraging their advantages of “multiple targets, multi-levels, and holistic regulation,” are widely applied to modulate mitochondrial function and alleviate mitochondrial dysfunction, emerging as a novel therapeutic direction for OP prevention and treatment. This review summarizes the core mechanisms by which mitochondrial dysfunction drives OP, and systematically catalogs CBD-derived natural chemical metabolites (e.g., resveratrol) and classic formulations (e.g., Zuogui Pill) that target and ameliorate mitochondrial dysfunction. This review aims to establish a “CBD-mitochondrial-bone” research paradigm, highlight the most promising CBD candidates for alleviating mitochondrial dysfunction, and provide a theoretical basis and novel insights for developing precision treatment strategies for OP based on ameliorating mitochondrial dysfunction.

## 1 Introduction

Osteoporosis (OP) is a systemic metabolic disorder characterized by reduced bone mass, impaired bone microarchitecture, increased bone fragility, and susceptibility to fractures (Reid and Billington, 2022). Under physiological conditions, osteoblasts (OBs) and osteoclasts (OCs) maintain a dynamic equilibrium of bone formation and resorption in the human body. However, this equilibrium is disrupted during aging, estrogen depletion, oxidative stress, and chronic inflammation, primarily manifested as an imbalance in the activity ratio between OBs and OCs (Sharma et al., 2018). Additionally, accelerated senescence of bone marrow mesenchymal stem cells (BMSCs) further contributes to dysregulation of bone lipid metabolism (Zhang et al., 2024a). Analysis indicates a global OP prevalence of approximately 18.3%, with women exhibiting significantly higher rates than men (Salari et al., 2021). Although numerous OP therapeutic agents exist clinically—including parathyroid hormone and its analogues, selective estrogen receptor modulators, calcitonin-like peptides, and bisphosphonates—no targeted treatment currently restores bone homeostasis equilibrium. Therefore, exploring its underlying pathogenesis is particularly imperative.

**FIGURE 1:**
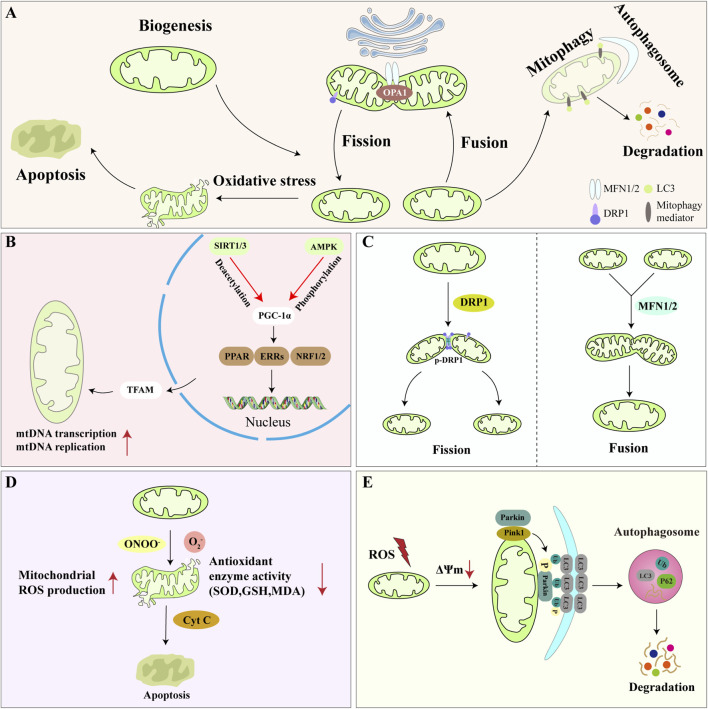
Mechanisms of mitochondrial dysfunction. **(A)** Overview of mitochondrial dysfunction; **(B)** During mitochondrial biogenesis, SIRT1/3 and AMPK activate PGC-1α through deacetylation and phosphorylation, respectively, promoting mtDNA transcription and replication via TFAM; **(C)** Mitochondrial fusion and fission depend on DRP1 and MFN1/2, respectively, to maintain mitochondrial number homeostasis; **(D)** Under OS, mitochondrial antioxidant enzymes and molecules (SOD, GSH) exhibit reduced activity, leading to increased Cyt c release and promoting inflammation and apoptosis; **(E)** ROS induce mitochondrial membrane potential (ΔΨm) decline and stimulate Parkin/PINK1 signaling pathway activation, resulting in autophagosome formation and ultimately triggering mitophagy and degradation.

Mitochondria are organelles possessing unique mitochondrial DNA (mtDNA). By generating adenosine triphosphate (ATP), they fulfill the energy demands of cells and are hailed as the “powerhouse” of the cell (Garbincius and Elrod, 2022; Peng et al., 2024). More importantly, mitochondria continuously influence cellular processes such as differentiation and apoptosis by participating in physiological events like oxidative stress and intracellular signaling. Recent studies (Pickles et al., 2018) reveal that mitochondrial dysfunction has emerged as a key factor in OP. Under physiological conditions, bone cells are regulated by mitochondrial fusion, fission, mitophagy, and transport processes, forming the “Mitochondria-bone” biological axis. This axis balances OBs and OCs functions by maintaining mitochondrial homeostasis (Yan et al., 2023). Mitochondrial biogenesis supplements new mitochondria to ensure energy metabolism; mitochondrial dynamics repair or eliminate damaged mitochondria through fusion and fission; mitochondrial oxidative stress increases intracellular reactive oxygen species (ROS) levels, disrupting mitochondrial structure; mitophagy selectively removes damaged and dysfunctional mitochondria (Figure 1). By ameliorating mitochondrial dysfunction, mitochondrial quantity and quality are strictly monitored to maintain bone homeostasis.

Chinese botanical drugs (CBDs) are rich in natural chemical metabolites and have been extensively studied in the clinical management of OP (Li K. et al., 2023). Based on the traditional Chinese medicine principle of “preventing disease before it occurs,” CBD offers multifaceted advantages in preventing and treating OP. CBD therapy improves pathological states through multi-target, multi-step, and multi-level approaches, better adapting to the complexity of OP. Modern research confirms that CBD-derived natural chemical metabolites and formulations can regulate mitochondrial function and alleviate mitochondrial dysfunction through specific pathways, thereby exerting therapeutic effects on OP. Although existing evidence supports the potential of CBD to ameliorate mitochondrial dysfunction, large-scale clinical trials remain relatively scarce. Furthermore, the precise mechanisms underlying CBD’s treatment of OP remain unclear, making it difficult to definitively establish its efficacy and active constituents. This review aims to elucidate the relationship between mitochondrial dysfunction and OP, and to outline the research progress on how CBD (including natural chemical metabolites and formulations) prevents and treats OP by alleviating mitochondrial dysfunction. It provides insights for the clinical development of new therapies for OP.

A total of 303 articles were initially identified from PubMed (n = 15), Web of Science (n = 20), Embase (n = 70), ScienceDirect (n = 119), and China National Knowledge Infrastructure (CNKI) (n = 79) using key words such as “Mitochondria”, “Osteoporosis”, and “Chinese botanical drug”, focusing primarily on studies published within the last 10 years. A limited number of older references were also included. The articles were then screened according to their relevance to the research topic and full-text availability. After applying these criteria, a final selection of 39 articles was made. For a more detailed search process, see Supplementary Material 1.

## 2 Mitochondrial structure and function

Mitochondria are double-membrane organelles composed of the outer mitochondrial membrane (OMM) and the inner mitochondrial membrane (IMM), whose structural specialization directly underpins their energy metabolism and signaling regulation functions—a core basis for the proliferation, differentiation, and functional exertion of bone cells (OBs, OCs, BMSCs) that rely on precise energy supply and signal transduction for bone homeostasis.

The OMM, composed mainly of lipids and proteins, is freely permeable to small molecules and ions, separating the mitochondrial interior from the rest of the cell and providing a platform for communication and interaction between mitochondria and other organelles (Pei et al., 2024). For BMSCs, the OMM mediates calcium ion (Ca^2+^) exchange and lipid metabolism signaling with the endoplasmic reticulum via mitochondria-associated endoplasmic reticulum membranes (MAMs)—a critical process for BMSC fate determination (osteogenic vs. adipogenic differentiation). In addition, the OMM controls the active transport of substances between mitochondria and the cytoplasm, and its integrity is essential for preventing the release of pro-apoptotic factors [e.g., Cytochrome C (Cyt C)] (Gomkale et al., 2021; Li Y. et al., 2025). For OBs and OCs, OMM damage triggers apoptotic pathways, directly inhibiting osteogenic differentiation and excessive bone resorption, respectively, which is an early event in mitochondrial dysfunction-induced bone cell damage in OP.

Unlike the OMM, the IMM is impermeable to most molecules and ions and requires specific membrane transporter proteins to transport larger molecules and ions across the membrane. A variety of biochemical reactions take place in the IMM (Strubel et al., 2022), with the presence of the electron transport chain (ETC.), ATP synthase, adenosine diphosphate (ADP)/ATP translocase, and many other membrane transport systems (Cadonic et al., 2016). OCs rely on a massive ATP supply for the formation of bone-resorbing ruffled borders, and the IMM’s OXPHOS activity is the primary source of ATP for OC-mediated bone resorption; OBs require continuous ATP support for osteoid synthesis and mineralization, and the efficiency of IMM OXPHOS directly determines the rate of osteogenic differentiation. The IMM folds into tubular or lamellar cristae (Rigotto and Basso, 2019), which expand the inner membrane surface area to maximize ATP synthesis—the cristae structure of OCs is more developed than that of OBs to adapt to the high energy demand of bone resorption, while the cristae integrity of aged or estrogen-deficient BMSCs/OBs is damaged, leading to reduced ATP production and impaired osteogenic potential.

The IMM, together with the OMM, divides the mitochondria into two major subspaces: an inner space, the mitochondrial matrix, and a small region between the OMM and IMM, the mitochondrial membrane space (IMS). The mitochondrial matrix is the main site of aerobic oxidation, and various enzymes involved in biochemical reactions such as the tricarboxylic acid cycle, fatty acid oxidation, and amino acid degradation are present in the mitochondrial matrix (Guedouari et al., 2021). For BMSCs, mitochondrial matrix fatty acid oxidation metabolism regulates the switch of osteogenic/adipogenic differentiation—enhanced fatty acid oxidation promotes osteogenic differentiation, while its inhibition leads to adipogenic differentiation and bone marrow fat accumulation, a typical feature of OP. Furthermore, mitochondria are semi-autonomous organelles containing double-stranded circular DNA (mtDNA), which is located in the mitochondrial matrix along with ribosomes, allowing for relatively independent replication, transcription, and translation of genetic information (Čater and Bombek, 2022). mtDNA mutations or copy number reduction in bone cells are common in OP: damage to mtDNA in OBs leads to reduced expression of oxidative phosphorylation complexes and decreased ATP synthesis, whereas excessive mtDNA replication in OCs promotes excessive mitochondrial biogenesis and bone resorption activity. Notably, the coordinated interaction between nuclear DNA and mtDNA is essential for maintaining optimal mitochondrial function—this coordination is disrupted in senescent BMSCs, leading to impaired mitochondrial biogenesis and further inhibition of osteogenic differentiation, a key link between mitochondrial genetic regulation and OP pathogenesis (Gustafsson et al., 2016).

Overall, mitochondria are the core organelle regulating bone cell energy metabolism, signal transduction, and survival/apoptosis, and their structural integrity and functional stability are essential for maintaining the proliferation, differentiation, and normal function of BMSCs, OBs, and OCs. Mitochondrial structural damage (e.g., cristae fragmentation, OMM/IMM disruption) and functional disorders (e.g., OXPHOS impairment, mtROS overproduction, mtDNA mutation) in bone cells are the initial and core links of mitochondrial dysfunction, and directly trigger the occurrence and progression of OP (Cai et al., 2023; Picca et al., 2018; Rovira-Llopis et al., 2017). Therefore, maintaining the structural and functional homeostasis of bone cell mitochondria is a crucial step in protecting bone cell function and delaying OP onset and progression.

## 3 The role of mitochondrial dysfunction in OP

Mitochondrial function is regulated to maintain mitochondrial morphology and quantity to sustain the functional stability of the mitochondrial network system. It serves as a key mechanism coordinating various mitochondrial biological functions, including biogenesis, dynamics, oxidative stress, and mitophagy (Liu et al., 2021). Mitochondrial biogenesis primarily refers to the synthesis of new mitochondria, whose main role is to maintain mitochondrial stability. Mitochondrial dynamics, on the other hand, maintain the dynamic equilibrium of mitochondrial morphology and function through fusion and fission (Eisner et al., 2018). Excessive intracellular ROS may damage the IMM and mtDNA. To counter this, cells activate their antioxidant defense system—namely, mitochondrial oxidative stress—to ensure mitochondrial integrity. Furthermore, mitophagy ensures normal mitochondrial function by eliminating damaged mitochondria (Sun K. et al., 2020).

**FIGURE 2:**
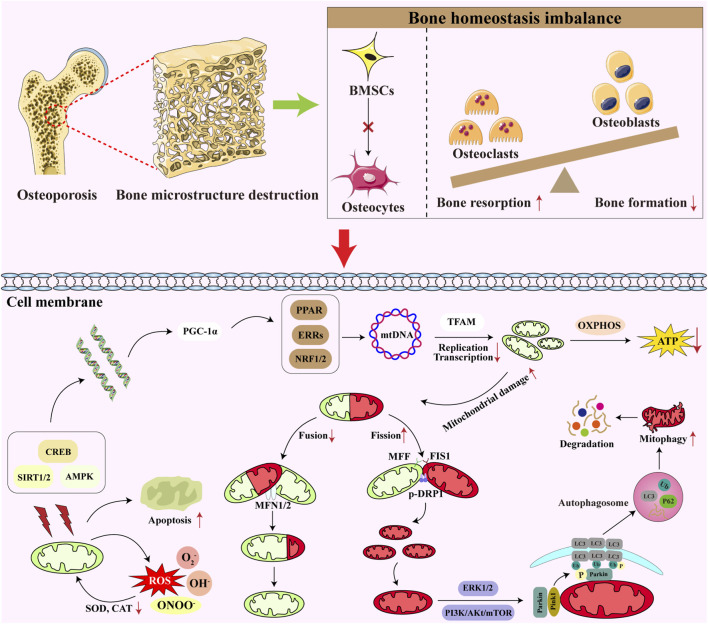
Mitochondrial dysfunction in OP. OP primarily manifests as a disruption of bone microstructure. At the cellular level, this is mainly characterized by the inhibition of osteogenic differentiation in BMSCs. Additionally, it involves an imbalance between OCs and OBs, leading to reduced bone formation and increased bone resorption. From a mitochondrial perspective, OP suppresses mitochondrial biogenesis, manifested by the inactivation of key regulators such as SIRT1/2 and PGC-1α. This ultimately reduces ATP synthesis, hindering bone formation. Furthermore, excessive ROS accumulation triggers mitochondrial oxidative stress, further increasing mitochondrial apoptosis and promoting bone cell death. Moreover, osteoporotic patients exhibit reduced mitochondrial fusion and increased fission. Through regulation of the Parkin/PINK1 signaling pathway, this promotes excessive mitophagy.

OP is a systemic skeletal disorder characterized by decreased bone mass, impaired bone microstructure, increased bone fragility, and susceptibility to fracture. At the cellular level, OP is caused by two main factors: a dysregulation between the lipogenic and osteogenic differentiation of senescent BMSCs and an imbalance between OB-mediated bone formation and OC-mediated bone resorption. Cellular senescence in the bone microenvironment plays an important role in the onset and development of OP, and local inflammation, oxidative stress, and metabolic-immune disorders can disrupt the balance of the bone microenvironment, ultimately leading to OP (Kimball et al., 2021; Li et al., 2022). Research (Das and Kale, 2021) indicates that metabolic alterations and mitochondrial dysfunction frequently accompany the progression of OP, while risk factors contributing to poor bone health are also prevalent in primary mitochondrial disorders (Gandhi et al., 2017). Progressive deterioration of mitochondrial dysfunction exacerbates the pathogenesis of OP (Figure 2). Unraveling the molecular mechanisms governing mitochondrial dysfunction is therefore essential for maintaining bone homeostasis and protecting the skeletal system from mitochondrial damage.

### 3.1 The role of mitochondrial biogenesis in OP

#### 3.1.1 Mitochondrial biogenesis

Mitochondrial biogenesis refers to the formation of new mitochondria, which is mainly regulated by nuclear DNA and mtDNA, and generates new mitochondria through the growth and division of existing mitochondria, in order to increase mitochondrial quantity and quality to adapt to energy metabolism (Popov, 2020). Impairment of mitochondrial biogenesis is often characterized by mitochondrial structural dysregulation, reduction of mtDNA, and decreased levels of biogenesis-related mRNA (Chodari et al., 2021). Peroxisome proliferator-activated receptor gamma coactivator 1-alpha (PGC-1α) is a transcriptional coactivator whose activity is precisely regulated by the upstream signaling pathway adenylate-activated protein kinase/silent information regulator 1 (AMPK/SIRT1). They are regarded as metabolic sensors regulating mitochondrial biogenesis, and their role in controlling mitochondrial respiration and ATP synthesis under energy-depleted conditions has been well established. It then further activates mitochondrial transcription factor A (TFAM) by regulating the activity of downstream nuclear respiratory factor 1/2 (Nrf1/2), thereby initiating the transcription and replication of mtDNA and completing mitochondrial biogenesis (Coppi et al., 2021). PGC-1α is closely related to the regulation of mitochondrial respiration and ATP synthesis, and can promote mitochondrial biosynthesis and reduce mitochondrial dysfunction (Ma et al., 2024). Various molecules form a network in PGC⁃1α mitochondrial biogenesis, and in OP-associated cells, molecules such as SIRT1/3, Nrf1/2, and estrogen-related receptor α (ERRα) have been demonstrated to interact with PGC⁃1α and participate in the regulation of mitochondrial biogenesis (Wang and Wang, 2013). These molecular interactions and regulatory networks play an important role in promoting mitochondrial biosynthesis, regulating mitochondrial respiration, and ATP synthesis.

#### 3.1.2 Regulation of mitochondrial biogenesis in OBs

The differentiation of BMSCs into osteocytes, as well as the proliferation and differentiation of OBs, are both accompanied by an upregulation of mitochondrial biogenesis (Forni et al., 2016). Researchers have found (Huang et al., 2017) that ERRα enhances mitochondrial function by increasing the expression of glutaminase in mitochondria during osteogenic differentiation of BMSCs. In BMSCs from aged mice, the expression of PGC-1α and ERRα is reduced, but the compensatory effect of ERRα rescues the osteogenic capacity of BMSCs. Similarly, PGC⁃1α expression is significantly reduced in BMSCs of ovariectomized (OVX) mice, and mRNA expression levels of key regulators of mitochondrial biogenesis (e.g., PGC⁃1α, TFAM) are low, and, conversely, upregulation of these transcription factors promotes mitochondrial biogenesis (Li, M. et al., 2023). It is known that SIRT1 and Nrf2-related signaling pathways enhance mitochondrial biogenesis in osteoprogenitor cells by acting on PGC-1α, thereby exerting anti-osteoporotic effects by alleviating mitochondrial dysfunction (Tang et al., 2020; Zhang et al., 2019b). Ma et al. (Ma et al., 2018) demonstrated that resveratrol activates PGC-1α by upregulating SIRT1 expression, thereby increasing mitochondrial ATP production, enhancing mitochondrial membrane potential, reducing mitochondrial ROS, and promoting mitochondrial biogenesis. This significantly elevated alkaline phosphatase (ALP), osteocalcin (OCN), osteopontin (OPN), and runt-related transcription factor 2 (Runx2) expression levels, thereby promoting osteogenic differentiation in MC3T3-E1 cells. Nrf2 similarly regulates mitochondrial function (Sies et al., 2017). PGC-1α, a key regulator of mitochondrial biogenesis, synergistically activates Nrf1/2 transcription to jointly regulate the expression of nuclear-encoded mitochondrial genes (e.g., TFAM, Cyt C, and mitochondrial complexes), which collectively govern mitochondrial biogenesis (Wenz, 2013). During osteogenic differentiation, enhanced mitochondrial biogenesis elevates Nrf2 activity, thereby promoting differentiation specificity by transcribing osteoblast-specific genes (Sánchez-de-Diego et al., 2021). Furthermore, studies (Ding et al., 2017) reveal that PGC-1α overexpression can suppress the reduction in mitochondrial density, membrane potential, and ALP activity induced by SIRT3 knockdown in OBs. Tang et al. (2020) demonstrated that NaB enhances mitochondrial redox homeostasis, energy metabolism, and mitochondrial antioxidant enzyme activity by regulating the Nrf2/GSK-3β signaling pathway and activating PGC-1α and TFAM. Furthermore, mitochondrial DNA polymerase γ (Polg) performs critical functions in mitochondrial DNA replication and repair within cells. Dobson et al. (2020) found that Polg mutations lead to decreased expression of mitochondrial respiratory chain proteins in OBs, subsequently causing mitochondrial dysfunction, accelerating bone loss, and inhibiting bone formation. Overall, PGC-1α, SIRT1/3, and Nrf2 may represent potential therapeutic targets for OP by ameliorating mitochondrial dysfunction.

#### 3.1.3 Regulation of mitochondrial biogenesis in OCs

Similar to PGC-1α, PGC-1β is a strong activator of mitochondrial biogenesis and regulates several aspects of energy metabolism. In the context of proliferation and differentiation, alterations in PGC-1β expression regulate mitochondrial biogenesis in OCs. Zhang et al. (2024b) observed that lentivirus-mediated silencing of the PGC-1β gene in RAW264.7 cells resulted in a reduction in the number of mature OCs. In addition, immunofluorescence analysis (Zhang H. et al., 2021) showed that PGC-1β expression was significantly reduced in the bone tissue of OVX mice, resulting in increased bone mass. The above *in vivo* and *in vitro* studies indicate that PGC-1β knockdown is a key gene for inhibiting mitochondrial biogenesis and osteoclast differentiation, thereby relieving mitochondrial dysfunction in OCs. Furthermore, studies (Zhang Y. et al., 2021) indicate that SIRT3 suppresses osteoclast differentiation and mitochondrial biogenesis by regulating the expression of AMPK, PGC-1β, and ERRα, thereby reducing mtDNA content and the expression of mitochondrial biosynthetic markers such as PGC-1α, TFAM, and SOD2. Peroxisome proliferation-activated receptor (PPAR) can promote osteoclast differentiation by indirectly inducing PGC-1β expression through downregulation of β-catenin protein levels and inhibition of c-Jun expression. On the other hand, PPARγ also induces ERRα expression and synergistically induces mitochondrial genes involved in fatty acid β-oxidation (β-FAO) and OXPHOS together with PGC-1β, enhancing mitochondrial biogenesis and promoting osteoclast differentiation and function (Wei et al., 2010). Receptor activator of NF-κB ligand (RANKL) stimulation enhances mtDNA levels and increases mitochondrial biogenesis in bone marrow macrophages in a dose-dependent manner. In contrast, activation of the CREB signaling pathway reduces PGC⁃1β levels to inhibit mitochondrial biogenesis and ameliorate homeostatic imbalance in bone (Yuan et al., 2022).

Overall, most of the current studies on the involvement of mitochondrial biogenesis in the regulation of OP are related to PGC-1α/β expression and activity. Therefore, how to regulate mitochondrial biogenesis to alleviate mitochondrial dysfunction and achieve therapeutic effects in OP still needs to be further explored in the future.

### 3.2 The role of mitochondrial dynamics and OP

#### 3.2.1 Mitochondrial dynamics

The continuous movement and morphological changes of mitochondria form a dynamic, continuous network known as mitochondrial dynamics, which involves mitochondrial fusion and fission (Marín-García and Akhmedov, 2016; Mishra and Chan, 2016). Mitochondrial fission produces smaller organelles that maintain mitochondrial number, cell polarity, and help eliminate damaged mitochondria, while mitochondrial fusion promotes the exchange and attachment of mitochondrial contents to provide sufficient energy to mitigate oxidative damage and maintain membrane potential (Ren et al., 2020). The dynamic balance between fission and fusion is critical for maintaining optimal mitochondrial function and meeting specific cellular energy metabolism requirements (Chan, 2012).

Three factors are involved in the regulation of mitochondrial fusion: mitofusin1 (MFN1), mitofusin2 (MFN2), and optic atrophy 1 (OPA1), which are mainly localized to the OMM and are anchored to the OMM by their termini (Chandhok et al., 2018). MFN1/2 mediate the fusion of the outer mitochondrial membrane, and they facilitate the fusion of neighboring OMM to form a continuous outer membrane structure, which contributes to the maintenance of overall mitochondrial morphology and function (Harrington et al., 2023). OPA1 is localized in the IMM and mediates fusion of the inner membrane through its specific structural domains. Endosomal fusion is regulated by multiple allelic variants of OPA1, which are involved in the formation of mitochondrial endosomal cristae and the maintenance of endosomal integrity (Del Dotto et al., 2018). When mitochondrial function is impaired, MFN1/2 anchors to the neighboring OMM via its carboxyl terminus and releases energy by hydrolysis of ATP to cause fusion of the OMM, and then anchors to the neighboring IMM via OPA1 to stabilize and maintain the mitochondrial morphology, cristae structure, and endomembrane integrity, which are essential for normal cellular function and metabolic processes.

Two factors are involved in the regulation of mitochondrial fission: mitochondrial fission protein 1 (FIS1) and dynamin-related protein 1 (Drp1), which are located in the OMM and cytoplasm, respectively. Under stress conditions, FIS1, mitochondrial fission factor (MFF), and mitochondrial elongation factor 1/2 (MIEF1/2) on the OMM together recruit DRP1 from the cytoplasm to the mitochondrial surface, where it is assembled into a highly oligomeric cyclic complex that encapsulates the mitochondria and mediates mitochondrial fission through its guanosine triphosphate (GTP) enzyme activity. activity to mediate mitochondrial division (Adebayo et al., 2021; Kalia et al., 2018; Yu et al., 2019; Yu et al., 2021). However, in addition to the mediation and binding of Drp1, mitochondrial fission is also affected by lysosomes (Wong et al., 2018), actin filaments, and actin regulators (Manor et al., 2015). According to a related study (Guo et al., 2013), inhibition of Drp1 activity reduces the degradation of lysosomes and thus promotes mitochondrial division. From the above, it can be seen that various mediators interfere with the activity of mitochondrial proteins, and a variety of biochemical effects occur to promote mitochondrial division.

#### 3.2.2 Regulation of mitochondrial dynamics in OBs

Mitochondrial dynamics play an important role in the pathogenesis of OP. When the balance between mitochondrial fusion and fission is disturbed, it induces mitochondrial dysfunction, affects the activity of OBs and OCs and accelerates the onset and progression of OP. Drp1, as a central factor in mitosis, plays a crucial role in maintaining osteogenic function. Research (Zhang et al., 2017) indicates that tumor necrosis factor-α (TNF-α) and interleukin-6 (IL-6) mediate increased Drp1 levels, leading to excessive mitochondrial segmentation and ROS accumulation. This disrupts mitochondrial membrane potential, triggers mitochondrial vesiculation and fragmentation, ultimately impairing mitochondrial function and suppressing OB activity. In contrast, the use of the oxidant N-acetylcysteine (NAC) or the mitochondrial fragmentation inhibitor Mdivi-1 counteracted the reduction in OB proliferation and differentiation caused by Drp1-promoted mitochondrial fragmentation, thereby ameliorating inflammation-induced osteogenic dysfunction and mitochondrial dysfunction. Furthermore, a high-glucose environment alters mitochondrial dynamics in OBs, leading to decreased Drp1 expression, increased fusion mitochondria, reduced fragmented mitochondria, and diminished mitochondrial biogenesis. This impairs migration and chemotaxis of OBs (Pahwa et al., 2020). It can be seen that mitochondrial dynamics play an important role in the regulation of proliferation and differentiation of OBs through the regulation of mitochondrial function and alleviation of mitochondrial dysfunction.

#### 3.2.3 Regulation of mitochondrial dynamics in OCs

Mitochondrial dynamics have also been found to influence osteoclast differentiation. Knockdown of MFN1/2 in OCs increased bone mass in mice, and MFN2 not only promoted mitochondrial fusion (Nicassio et al., 2017) but also activated nuclear factor of activated T-cells c1(NFATc1) expression (Yang et al., 2014), whereas overexpression of MFN2 reversed osteoclast defects in OCs, suggesting that mitochondrial dynamics have an important effect on bone resorption (Ballard et al., 2020b). RANKL has been reported to regulate the expression of Drp1 and its receptor proteins FIS1, Mid49, and Mid51. In addition, glycogen synthase kinase-3β (GSK-3β) inhibitors have been shown to increase Drp1 expression and promote osteoclast differentiation. These results suggest that the RANKL/GSK-3β/Drp1 axis promotes osteoclast differentiation. Drp1 enhances the differentiation of OCs by promoting the increase of c⁃fos/NFATc1 axis during osteoclast differentiation. Inhibition of Drp1 inhibits lipopolysaccharide-induced osteoclast differentiation and attenuates OVX-induced bone loss *in vivo* in a cranial model (Jeong et al., 2021).

In summary, mitochondrial fusion and fission are important for OBs and OCs activity, and intervening in mitochondrial dynamics to relieve mitochondrial dysfunction is a potential strategy for the treatment of OP.

### 3.3 The role of mitochondrial oxidative stress in OP

#### 3.3.1 Mitochondrial oxidative stress

Mitochondria perform vital physiological functions, including OXPHOS, electron transport, and energy metabolism. They serve as the primary source of oxidative stress within cells and a key target for apoptosis. Oxidative stress refers to the disruption of the dynamic equilibrium between ROS production and scavenging, leading to damage of critical cellular components such as proteins, lipids, and DNA, and severely impairing cellular structure and function (Ferramosca et al., 2017). Oxidative stress manifests as inhibition of mitochondrial respiratory enzyme activity and slowed, ETC., transfer, leading to mitochondrial dysfunction and excessive ROS accumulation within mitochondria (Biary et al., 2011). During OXPHOS, oxygen is incompletely reduced to superoxide radicals (O_2_^−^) by Cyt C oxidase. These superoxide radicals (O_2_^−^) are converted to H_2_O_2_ by mitochondrial superoxide dismutase and released into the intermembrane space and cytoplasm, thereby generating ROS (Zorov et al., 2014a). Simultaneously, excess superoxide anions (O_2_^−^) can deplete the antioxidant capacity mediated by NADPH, leading to further ROS release from mitochondria. Excessive mtROS may trigger the opening of the mitochondrial permeability transition pore (mPTP), a channel in the IMM. When activated, this pore leads to a reduction in the mitochondrial membrane potential (Bronner and O’Riordan, 2016). Concurrently, mtROS induces mitochondrial membrane lipid peroxidation, protein inactivation, and mtDNA mutations, further impairing, ETC., function and exacerbating ROS leakage. This creates a vicious cycle of “ROS accumulation-mitochondrial damage” (Tatjana et al., 2024). To prevent further cellular damage, the organelles mitigate the trouble by neutralizing superoxide through the concerted actions of Mn-SOD, catalase (CAT), and glutathione peroxidase (GPx), thereby preserving cellular homeostasis (Ismail et al., 2019). A mounting body of evidence (Zorov et al., 2014b) indicates that mitochondrial dysfunction exerts a pivotal function in mtROS-mediated redox imbalance. Mitochondria have been shown to limit excessive mtROS production and maintain redox balance by phagocytosing aged and damaged mitochondria. For instance, research (Ning et al., 2021) indicates that long-term exposure to PM2.5 can drive excessive mitochondrial ROS and undermine the mitochondria’s ability to engage in mitophagy, leading to a redox imbalance. On the other side, adding antioxidants that target mitochondria, like coenzyme Q10, can enhance mitochondrial mitophagy activity and curb oxidative harm by limiting ROS buildup. Additionally, agents that inhibit pathways such as AMPK, MAPK, and Nrf2 have been shown to promote ROS clearance by boosting how actively mitochondria participate in phagocytosis (Chen et al., 2024; Franci et al., 2022). In turn, this supports the upkeep of mitochondrial redox equilibrium. Notably, excessive mtROS may also induce the release of apoptotic proteins, further exacerbating mitochondrial dysfunction.

#### 3.3.2 Regulation of mitochondrial oxidative stress in OBs

Recent studies have revealed that mitochondria serve as crucial energy metabolism components in OBs, and mitochondrial dysfunction induced by oxidative stress directly contributes to the activation of intrinsic apoptosis pathways in OBs. ROS overload causes free radical attacks on the phospholipid bilayer, leading to mitochondrial membrane depolarization. This opens the mitochondrial membrane pores, resulting in the loss of mitochondrial membrane potential. During this process, increased permeability of the mitochondrial membrane allows the apoptotic factor Cyt C into the cytoplasm and activates caspase-9, marking the primary initial step in the intrinsic apoptotic pathway. Activated caspase-9 sequentially activates downstream pro-apoptotic factors such as caspase-3 and caspase-7, ultimately inducing cell death. Furthermore, activation of the caspase signaling is tightly regulated by the expression of various modulators (Bock and Tait, 2020). Among these, members of the B-cell lymphoma-2 (Bcl-2) family—comprising pro-apoptotic and anti-apoptotic proteins—play a pivotal role in determining the progression of the intrinsic apoptotic pathway. As a representative pro-apoptotic protein, Bcl-2-associated X protein (Bax) localizes to the OMM, inducing Cyt C release by promoting mitochondrial permeability transition or impairing the barrier function of the outer membrane. Conversely, the anti-apoptotic protein Bcl-2 is essential for maintaining mitochondrial permeability and membrane barrier stability to inhibit the release of pro-apoptotic factors. Therefore, the equilibrium between pro-apoptotic and anti-apoptotic Bcl-2 family proteins is decisive in inducing the activation of the caspase signaling, which is the decisive factor in initiating the intracellular apoptosis pathway (Bock and Tait, 2020; Edlich et al., 2011). Cai et al. (Cai et al., 2013) reported that co-culture with TNF-α increased cellular ROS and malondialdehyde (MDA) production, elevated NADPH oxidase activity, and showed a trend toward increased mitochondrial ROS levels, while ATP synthesis declined. Additionally, antioxidant enzyme activity (including superoxide dismutase and catalase) was suppressed, negatively impacting mitochondrial function and aggravating mitochondrial dysfunction. Ho et al. (2009) found that exposure of human osteosarcoma MG-63 cells to hydrogen peroxide significantly increased cellular oxidative stress, thereby inducing enhanced apoptosis. In summary, hydrogen peroxide reduced mitochondrial membrane potential, elevated levels of Cyt C and caspase-3, and suppressed Bcl-2 mRNA and protein expression. These findings demonstrate the pivotal role of mitochondrial ROS in mediating mitochondrial dysfunction and promoting apoptosis of OBs.

#### 3.3.3 Regulation of mitochondrial oxidative stress in OCs

ROS accumulation, as a key signaling factor of mitochondrial oxidative stress, also participates in osteoclast differentiation. Studies by Baek et al. (2010); Fraser et al. (1996) revealed that H_2_O_2_ promotes OC proliferation and differentiation in mouse skull bone, BMSCs, and human bone marrow cells. Bartell et al. (2014) observed that in H_2_O_2_-treated mouse cells, reduced intracellular H_2_O_2_ levels corresponded with decreased osteoclast differentiation and maturation. It has been demonstrated that Nrf2 inhibits osteoclast differentiation (Lee et al., 2005). Srinivasan et al. (Srinivasan et al., 2010) observed a significant reduction in the number of differentiated and mature mouse OCs following the addition of antioxidants. Exogenous antioxidants demonstrated a marked ability to suppress the elevated ROS levels during osteoclast differentiation induced by Nrf2 deficiency, thereby inhibiting Nrf2-deficient osteoclast differentiation. Increased ROS levels lead to c-fos phosphorylation, which in turn stimulates OC development. Nrf2 deficiency promotes osteoclast differentiation, while c-fos inhibition blocks osteoclast differentiation (Agidigbi and Kim, 2019; Yang et al., 2023). They also found elevated intracellular NF-κB activity under hypoxic conditions, suggesting ROS may influence osteoclast differentiation via the RANK/RANKL signaling pathway. Ishii et al. (Ishii et al., 2009b) demonstrated ROS involvement in PGC-1β transcription. PGC-1β positively and negatively regulates ROS production, thereby promoting OC generation. Furthermore, Kim et al. (2013) investigated mitochondrial calcium signaling and found that knocking out the mouse endoplasmic reticulum transmembrane protein 64 (ERTP64) resulted in weakened cytoplasmic calcium oscillations, reduced ROS levels, and inhibited bone resorption. This suggests that calcium signaling may also represent one pathway through which ROS modulates osteoclast differentiation and induces mitochondrial dysfunction. The aforementioned studies indicate the existence of a complex regulatory network between ROS and osteoclast differentiation, with specific mechanisms potentially involving pathways such as RANK/RANKL, PGC-1β, and calcium signaling.

### 3.4 The role of mitophagy in OP

#### 3.4.1 Mitophagy

Mitophagy is a specialized form of autophagy that selectively degrades damaged mitochondria and promotes mitochondrial metabolism, specifically targeting the degradation of impaired or dysfunctional mitochondria (Goldman et al., 2010). Based on differences in the recognition mechanisms between phagophores and damaged mitochondria, it can be classified into ubiquitin-dependent and receptor-dependent mitophagy (Quiles and Gustafsson, 2020).

The ubiquitin-dependent pathway is primarily regulated by PTEN-induced kinase 1 (PINK1) and the E3 ubiquitin-protein ligase Parkin. In this mechanism, mitochondrial damage disrupts PINK1’s entry into the IMM. This leads to stable accumulation of PINK1 on the cytoplasmic face of the OMM, where it recruits and activates Parkin. The spatial conformation of the Parkin protease changes, converting it into an active E3 ubiquitin ligase. This ligase then ubiquitinates mitochondrial proteins. PINK1 interacts with Parkin, jointly regulating the mitophagy process to maintain mitochondrial quality (Ashrafi and Schwarz, 2013; Lazarou et al., 2015). Furthermore, beyond the PINK1/Parkin signaling pathway, PINK1 can also directly recruit autophagy receptor proteins such as Bcl-2 interacting protein 3 (BNIP3), its homolog NIP3-like protein X (NIX), and FUN14 domain-containing protein 1 (FUNDC1) to mitochondria. These receptor proteins recruit microtubule-associated protein light chain-3 (LC3) to autophagosomes. Microtubule-associated protein 1A/1B light chain 3 (LC3) is a key protein in autophagy that promotes mitophagy by targeting ubiquitin-labeled mitochondria to autophagosomes (Song et al., 2021).

Receptor-dependent mitophagy is mediated by mitophagy-related receptor 1 (MARR1), which contains long inverted repeat (LIR) sequences on the OMM. Under hypoxic conditions, BNIP3 and NIP3-like protein X (NIX) can bind to BCL-2 family proteins, activating mitophagy by inhibiting the mammalian target of rapamycin (mTOR) or regulating ROS production. The N-terminus of BNIP3 possesses an LIR sequence that recognizes LC3 and binds directly to it, thereby being recognized by phagophores and inducing mitophagy. Beyond the BNIP3/NIX pathway, FUNDC1 similarly localizes to the mitochondrial outer membrane. It directly interacts with LC3 via its LIR domain, thereby directly inducing mitophagy (Onishi et al., 2021). In summary, mitophagy plays a crucial role in maintaining intracellular homeostasis under both physiological and pathological conditions. Physiologically, it facilitates the clearance of damaged mitochondria, supporting energy metabolism and cellular function. Pathologically, modulating mitophagy through pharmacological or other interventions can help restore mitochondrial homeostasis within cells and alleviate cellular dysfunction.

#### 3.4.2 Regulation of mitophagy in OBs

Increasing evidence indicates that abnormal mitophagy plays a pivotal role in bone metabolic disorders. Lee et al. (Lee et al., 2021) found PINK1 to be downregulated in OP patients, and PINK1 deficiency exacerbated bone loss in OVX mice. This manifested as reduced expression of osteoblastic markers, including ALP, bone-specific protein (BSP), OCN, and OPN, indicating PINK1 promotes OB proliferation and differentiation. Concurrently, they observed upregulation of DRP1 and FIS1 while downregulation of MFN1 during osteogenic differentiation. Reduced PINK1 expression impaired mitochondrial homeostasis and function, leading to excessive ROS production and abnormal calcium uptake, thereby inhibiting osteogenic differentiation. Li W. et al. (2023a) found that PINK1/Parkin-mediated mitophagy reduces plasma advanced oxidative protein products (AOPP) levels and inhibits AOPP-induced apoptosis of OBs, thereby improving AOPP accumulation-related bone loss, microstructural disruption, and bone mineral density decline. Inhibition of mitophagy exacerbates the loss of osteogenic capacity and bone loss in diabetic mice. Wang et al. (2021) found that mitochondrial ferritin deficiency induces mitophagy via the PINK1/Parkin signaling pathway, thereby exacerbating ferroptosis in OBs under high-glucose conditions. This series of studies further supports the importance of the PINK1/Parkin signaling pathway for the function and survival of OBs, emphasizing the critical role of mitophagy in this process.

HIF-1α is widely expressed in human and mammalian cells, serving as a primary regulator of numerous hypoxia-inducible genes and playing a crucial role in both innate and adaptive immunity under hypoxic conditions (Zhao Y. et al., 2024). BNIP3 is a downstream target of HIF-1α and is regulated by HIF-1α (Tang et al., 2021). Xu et al. (2021) found that both BNIP3 and HIF-1α are upregulated in hypoxic environments, while their expression is downregulated upon exposure to glucocorticoids. Furthermore, under hypoxic conditions, overexpression of HIF-1α can inhibit glucocorticoid-induced cell death. This confirms that HIF-1α overexpression can mitigate the glucocorticoid-induced suppression of hypoxia-induced mitophagy-associated proteins through its downstream marker BNIP3, thereby protecting OBs from apoptosis.

In summary, mitophagy maintains the number of mitochondria within cells, ensuring mitochondrial function, thereby promoting proliferation and differentiation while protecting OBs from apoptosis. However, the role of mitophagy in OBs remains controversial. Excessive mitophagy can reduce mitochondrial quality, inhibit mitochondrial function, and lead to decreased proliferation and differentiation of OBs. For instance (Zhao et al., 2020), magnesium transporter non-imprinted in Prader-Willi/Angelman syndrome region protein 2 (NIPA2) counteracts PINK1/Parkin-mediated mitophagy in OBs by inhibiting the PGC-1/FoxO3a signaling pathway, thereby restoring osteogenic differentiation capacity impaired by high-glucose-induced excessive mitophagy.

#### 3.4.3 Regulation of mitophagy in OCs

Recent studies indicate that mitophagy plays a crucial role in osteoclast differentiation. Similar to OBs, the PINK1/Parkin signaling pathway is key to regulating mitophagy in OCs. Jang et al. (2023) found that PINK1 deficiency promotes the accumulation of damaged mitochondria, leading to increased ROS production. This enhances NFATc1 nuclear translocation, thereby increasing OC activity and promoting bone resorption. Osteoprotegerin (OPG), a member of the TNF receptor superfamily, inhibits osteoclast differentiation and activation while promoting apoptosis. Studies (Wang et al., 2020) indicate OPG significantly enhances mitophagy of OCs via the PINK1/Parkin signaling pathway.

MicroRNAs (miRNAs) are a class of endogenous small non-coding RNA molecules that play a crucial role in bone remodeling and bone metabolic diseases. miR-181a is a miRNA involved in multiple biological processes. miR-181a can suppress OPG protein expression and promote osteoclast differentiation by targeting OPG mRNA and inhibiting its translation (Gao and Shao, 2017). Furthermore, study (Sun Y. et al., 2020) indicates that miR-181a can influence relevant signaling pathways by targeting specific genes. For example, miR-181a upregulation inverts the extracellular regulated protein kinases (ERK) pathway by suppressing TNF-related apoptosis-inducing ligand (TRAIL) in intervertebral disc degeneration (IDD) mice. Conversely, Song et al. (2019) demonstrated that miR-181a overexpression downregulates activation of the MEK/ERK/NF-κB signaling pathway. Zhu et al. (2022) found that upregulating miR-181a expression targets Parkin downregulation, thereby reducing mitophagy, promoting OC survival, and consequently affecting bone remodeling homeostasis.

SIRT1 is the primary mitochondrial protein deacetylase and a key regulator of mitophagy, influencing multiple pathways that affect mitophagy within cells. Research (Jin et al., 2024) has demonstrated that modulating SIRT1 can regulate bone mass, with SIRT1 overexpression preventing age-related bone loss. SIRT3 regulates mitophagy of OCs by mediating PINK1 deacetylation, thereby promoting osteoclast differentiation. The SIRT3 inhibitor LC-0296 partially suppresses OC function, attenuating estrogen deficiency- or age-induced increases in bone resorption and bone mass reduction, thereby preventing OP (Ling et al., 2021).

In summary, mitophagy plays a crucial role in the formation and survival of OCs, and its mechanisms in relation to bone homeostasis warrant further in-depth investigation.

The core mechanisms and key targets by which these four aspects of mitochondrial dysfunction (biogenesis, dynamics, oxidative stress, and mitophagy) regulate osteoblasts and osteoclasts are systematically summarized in Table 1.

**TABLE 1:** Mechanism of mitochondria regulating OP.

Modulatory mechanism of mitochondria	Targets	Experimental models	Main results	References
Mitochondrial biogenesis	PGC-1α	Aged PGC1α-deficient mice	Upregulation of PGC-1α promotes the osteogenic differentiation of BMSCs and enhances the proliferation of OBs	Buccoliero et al. (2021), Graziana et al. (2018)
	PGC-1β	PGC-1β knockout OCs PGC-1β knockout mice	Downregulation of PGC-1β expression inhibited mitochondrial biogenesis and differentiation of OCs	Ishii et al. (2009a)
	Nrf2, SIRT1	Diabetic mice	Activated Nrf2 and SIRT1 enhance mitochondrial biogenesis and promote proliferation and differentiation of OBs	Zhang et al. (2019a)
Mitochondrial fusion	MFN2	MFN1 and MFN2 double conditional knockout mouse OC precursors	Enhanced MFN2 expression promoted bone formation and inhibited osteoclast precursor differentiation	Ballard et al. (2020a)
	OPA1	H_2_O_2_-treated MC3T3E1 cells	Upregulated OPA1 and reduced apoptosis of MC3T3-E1 cells	Cai et al. (2019)
Mitochondrial fission	Drp1	H_2_O_2_-treated Sao-2 cells	Inhibited Drp1-mediated mitochondrial fission and promoted osteogenic differentiation	Gan et al. (2015)
Mitochondrial oxidative stress	Nrf2	Dexamethasone (Dex)-treated MC3T3-E1 cells	Activated the Nrf2 pathway, reduced oxidative stress levels, and inhibited apoptosis of OBs	Chen, L. et al. (2020a)
	ROS	Dex-treated MC3T3-E1 cells	Reduced ROS levels, delayed BMSCs aging, promoted BMSCs osteogenic differentiation, and inhibited osteoclast differentiation	An et al. (2019), Chen M et al. (2022), Li et al. (2017), Zhen et al. (2014)
	SIRT3, AMPK	AGEs-induced BMSCs; OVX rats	Promoted the osteogenic differentiation of BMSCs and influenced the differentiation of OBs	Chen, L. et al. (2020b), Guo et al. (2021)
Mitophagy	PINK1/Parkin	Advanced oxidation protein products (AOPPs)-treated MC5T3-E1 cells	Activated Mitophagy and inhibited apoptosis of OBs	Li, W. et al. (2023b)
	PI3K/Akt/mTOR	H_2_O_2_-treated MC3T3E1 cells	Induced mitophagy, promoted BMSCs differentiation, and inhibited apoptosis of OBs	Zhao et al. (2022)
	ERK1/2	MC3T3E1 cells	Activated mitophagy and inhibited apoptosis of OBs	Sun et al. (2018)

## 4 CBDs for regulating the mitochondrial dysfunction in OP

**FIGURE 3:**
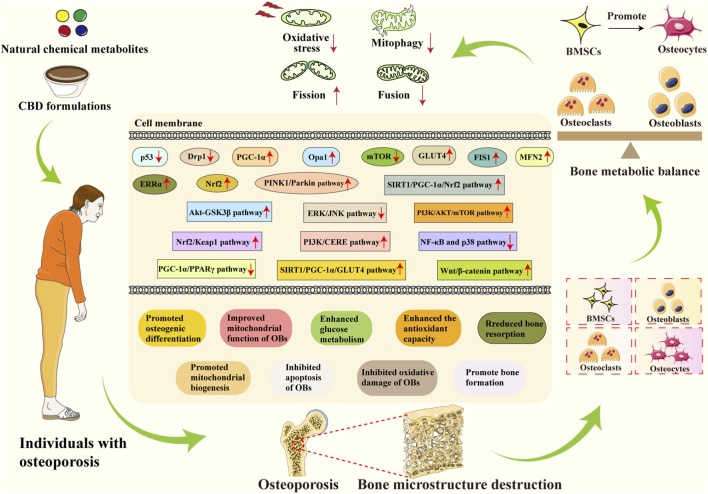
Schematic diagram of CBD improving OP by alleviating mitochondrial dysfunction. CBD improves bone metabolic imbalance in OP by regulating the balance between OBs and OCs and promoting the osteogenic differentiation of BMSCs. At the mitochondrial level, it alleviates mitochondrial oxidative stress, inhibits mitochondrial fission and mitophagy, and promotes mitochondrial fusion, thereby mitigating bone metabolic imbalance. Mechanistically, CBD enhances expression of key factors, including PGC-1α, ERRα, Nrf2, MFN2, and GLUT4 while reducing p53 and Drp1 expression. Furthermore, by promoting pathways like PINK1/Parkin, SIRT1/PGC-1α/GLUT4, and inhibiting NF-κB/p38 signaling pathways, they enhance osteogenic differentiation, suppress osteoclast differentiation, improve mitochondrial function, and enhance glucose metabolism capacity, ultimately ameliorating OP.

Chinese botanical drugs (CBDs), with their long history of clinical application and unique theoretical systems, offer distinct advantages in the prevention and treatment of complex metabolic diseases such as osteoporosis. Grounded in the holistic view of “syndrome differentiation and treatment,” CBDs exert multi-target, multi-level regulatory effects that align closely with the multifaceted nature of OP pathogenesis. Mitochondria represent a critical target for bone protection, and interventions based on alleviating mitochondrial dysfunction may constitute a key therapeutic strategy for OP. Mitochondrial modulators primarily exert their effects on bone metabolism by ameliorating mitochondrial dysfunction in OP. Numerous studies (Huh et al., 2015; Li, K. et al., 2023) have demonstrated that relevant CBD-derived natural chemical metabolites and formulations can restore bone morphology and function by regulating differentiation and metabolism of OBs and OCs, thereby slowing OP progression (Figure 3). The following sections summarize representative CBD-derived natural chemical metabolites and classical formulations that have been demonstrated to regulate pathways and alleviate mitochondrial dysfunction in preclinical and clinical studies.

### 4.1 Natural chemical metabolites for OP treatment by regulating mitochondrial dysfunction

#### 4.1.1 Natural chemical metabolites improve mitochondrial biogenesis to alleviate OP

Resveratrol, a polyphenolic compound derived from *Polygonum cuspidatum Sieb.et Zucc.*, enhances mtDNA content and mitochondrial quality by inducing AMPK phosphorylation and upregulating Nrf1/TFAM expression (Moon et al., 2020). Research indicates (Brockmueller et al., 2023) that it plays a prominent role in protecting bone health. Lipopolysaccharide (LPS) induces mitochondrial dysfunction in MC3T3-E1 cells, leading to increased mitochondrial superoxide and reduced levels of key osteogenic differentiation regulators such as ALP, OPN, OCN, Runx2, and other key regulators of osteogenic differentiation. As a potent antioxidant, resveratrol intervention increased ATP production in MC3T3-E1 cells while reducing superoxide generation, thereby promoting bone formation (Ma et al., 2018). Mitochondria are considered a target site for resveratrol, which promotes activation of SIRT1 and PGC-1α (Li S. et al., 2024). Ren et al. (2021) investigated the effects of anthocyanins and resveratrol on serum-starved hFOBs and found that anthocyanins upregulate SIRT1/3 and PGC-1α mRNA expression. This indicates anthocyanins promote mitochondrial biogenesis, primarily by downregulating apoptosis, Bcl-2 and Bax ratio, p53, and histone deacetylase 1 (HDAC1) expression. This enhances OB proliferation and differentiation, ultimately increasing bone formation and reducing bone loss. Therefore, resveratrol and anthocyanins effectively improve mitochondrial biogenesis through processes closely associated with SIRT1 and PGC-1α, which plays a crucial role in osteogenic differentiation in OP.

Liquiritigenin, a flavonone isolated from *Glycyrrhiza uralensis Fisch.*, is a potent antioxidant capable of scavenging free radicals in the body, alleviating oxidative stress, and protecting cells from damage (Carnovali et al., 2020). Methylglyoxal, a metabolic byproduct of glycolysis, exhibits elevated levels closely associated with OP. Suh et al. (2014) found that liquiritigenin mitigated its cytotoxicity in MC3T3-E1 cells, reduced ROS, superoxide, and lipid peroxide production, alleviated oxidative stress damage, and inhibited apoptosis of OCs. More importantly, liquiritigenin elevated PGC-1α expression, promoted aldehyde dehydrogenase I activity, and effectively inhibited mitochondrial membrane potential dissipation. This indicates that liquiritigenin can promote mitochondrial biogenesis, thereby enhancing osteogenic differentiation and ultimately achieving the goal of preventing and treating OP.

Genistein is a naturally occurring isoflavone compound primarily derived from *Glycine* max *(L.) Merr.*, exhibiting antioxidant and estrogen-regulating effects. Li, M. et al. (2023) demonstrated through *in vivo* experiments that genistein can regulate mitochondrial biogenesis via an ERRα-mediated mechanism, thereby mitigating senescence in BMSCs from OVX rats. Specifically, genistein reverses the decline in mitochondrial membrane potential and upregulates the expression of PGC-1α, Nrf1, key regulators of mitochondrial biogenesis. Furthermore, Genistein upregulates SIRT3 expression and promotes Parkin translocation to mitochondria, enhancing mitophagy. These effects are highly beneficial for alleviating OP.

1,2,3,4,6-Pentagalloylglucose (PGG) is an active component of *Schisandra chinensis (Turcz.) Baill. and Cynanchum otophyllum Schneid.*. *In vitro* and *in vivo* experiments (Chen T.-T. et al., 2020) indicate that the protective effect of PGG on OBs under oxidative stress conditions may be related to the activation of the Nrf2/HO-1 signaling pathway and the improvement of mitochondrial function, thereby preventing apoptosis of OBs. PGG reversed H_2_O_2_-induced increases in intracellular ROS production and mitochondrial damage, ultimately inhibiting apoptosis. Previous studies (Selim and Wojtovich, 2025) have demonstrated that ROS overexpression reduces mitochondrial membrane potential and decreases ATP production, leading to impaired mitochondrial function and structure while promoting apoptosis. Therefore, the Nrf2/HO-1 signaling pathway may represent a therapeutic target for PGG’s anti-OP effects.

*Morinda officinalis* polysaccharide (MOP) is one of the primary active components of *M. officinalis*, exhibiting excellent biological activity in combating OP and holding broad development prospects (Wu et al., 2024). Rong et al. (Rong et al., 2022) reported that MOP enhances bone density and serum Cu^2+^, Mg^2+^, and Fe^2+^ levels in OVX rats by inhibiting the PGC-1α/PPARγ signaling pathway. This leads to increased levels of SOD, GSH, and GPx, and MDA levels, thereby mitigating mitochondrial oxidative stress damage. It is speculated that sustained PGC-1α activation may exacerbate age-related trabecular bone loss and diminish the protective effects of this active compound.

Although the aforementioned studies consistently demonstrate that promoting osteoblast differentiation by activating the PGC-1α/SIRT1/Nrf2 axis through mitochondrial biogenesis is effective, the existing evidence still has significant limitations. First, most studies rely solely on *in vitro* cell models (such as MC3T3-E1 and BMSCs), lacking validation of drug efficacy in dynamic *in vivo* environments. For instance, while resveratrol significantly promotes mitochondrial biogenesis *in vitro*, its extremely low bioavailability *in vivo* raises doubts about achieving effective concentrations in bone tissue. Second, the selectivity of compound actions remains unclear: Genistein simultaneously modulates estrogen receptors and ERRα, and whether its anti-osteoporotic effects primarily stem from nuclear receptor regulation or direct mitochondrial action requires further separation and validation. Furthermore, some studies report conflicting mechanisms (e.g., MOP inhibits PGC-1α yet exerts protective effects), suggesting potential unidentified compensatory pathways or non-specific effects. Future research should integrate tissue-specific knockout animal models with pharmacokinetic analysis to clarify the direct targets and dose-response relationships of these compounds *in vivo*.

#### 4.1.2 Natural chemical metabolites improve mitochondrial dynamics to alleviate OP

Curcumin is a natural antioxidant isolated from the *Curcuma longa L.*, demonstrating potent preventive and therapeutic effects against OP (Li S. et al., 2025). Dai et al. (2017) found that curcumin reduces mTOR activity by activating the Akt-GSK3β signaling pathway, thereby increasing the mitochondrial membrane potential in OBs and mitigating oxidative stress-induced apoptosis. The study also revealed that GSK-3β inhibitors increase DRP1 expression, DRP1 being the primary protein regulating mitochondrial fission. This indicates that curcumin can modulate mitochondrial dynamics by intervening in the Akt/GSK3β signaling pathway, thereby inhibiting apoptosis and promoting osteoclast differentiation to achieve the effect of preventing OP.

Icariin is the primary active metabolite in *Epimedium brevicornu Maxim*. In a study by Yao et al. (2019), they found that ferrous ammonium citrate (FAC)-induced iron overload triggers an apoptotic pathway in BMSCs, while simultaneously suppressing Bcl-2 protein expression. They also observed that iron overload significantly depolarized mitochondrial membrane potential, increased ROS production, and markedly inhibited mitochondrial fusion and fission. Icariin intervention significantly attenuated iron overload-induced inactivation of the PI3K/AKT/mTOR signaling pathway and activation of the ERK1/2 and JNK signaling pathways. It increased the expression of mitochondrial fission protein FIS1 and fusion protein MFN2, thereby promoting the translocation of DRP1 and Cyt C from the cytoplasm to mitochondria. This enhanced the osteogenic differentiation and proliferation of BMSCs, reduced bone loss, and delayed OP.

Silibinin is a natural flavonoid lignan metabolite with potent antioxidant and mitochondrial protective properties. Research by Mao et al. (Mao et al., 2018) indicates that in OBs, silibinin directly downregulates receptor for advanced glycation end products (AGEs) expression, maintains the balance of osteogenic proteins L-OPA1 and S-OPA1, reduces mitochondrial oxidative stress and mitochondrial membrane potential, thereby significantly increasing ATP production. Additionally, silibinin improves abnormal mitochondrial fission/fusion events, inhibits apoptosis of OBs by enhancing mitochondrial dynamics, reduces osteocyte loss, and positively contributes to maintaining bone homeostasis, thereby moderately delaying OP progression.

In regulating mitochondrial dynamics, Curcumin, Icariin, and Silibinin exhibit distinct intervention strategies. Curcumin reduces excessive fission by inhibiting DRP1, thereby exerting anti-apoptotic effects (Dai et al., 2017); whereas Icariin acts bidirectionally, simultaneously upregulating the fission protein FIS1 and the fusion protein MFN2 to restore the kinetic imbalance caused by iron overload (Yao et al., 2019); Silibinin focuses on maintaining OPA1 subunit equilibrium to protect mitochondrial cristae architecture (Mao et al., 2018). However, the effective concentrations of these compounds vary significantly: Icariin requires concentrations as high as mM levels, which pharmacologically suggests potential non-specific mechanisms or the need for conversion to active metabolites *in vivo*, casting doubt on its direct monomeric drug ability. In contrast, Curcumin (30 μM) and Silibinin (100 μM) exhibit more commonly observed concentrations in the μM range. Future research should focus on the *in vivo* kinetic transformations of these compounds and how they precisely regulate the balance of fusion/fission proteins under different pathological conditions.

#### 4.1.3 Natural chemical metabolites improve mitochondrial oxidative stress to alleviate OP

Notoginsenoside R1 (NGR1) is an active metabolite in the *Panax notoginseng (Burkill) F. H. Chen ex C. H. Chow*, which has demonstrated positive mitochondrial protective effects. Research (Li et al., 2021) indicates that NGR1 restored levels and activity of osteogenic factors—including ALP, OCN, collagen I (COLI), and Runx2—in OBs damaged by oxidative stress. It significantly reactivated MMP, improved mitochondrial ROS production, increased ATP generation, and enhanced mtDNA replication. Furthermore, NGR1 significantly inhibited JNK phosphorylation, effectively blocking H_2_O_2_-induced JNK signaling activation. This indicates that NGR1 significantly attenuates oxidative stress-induced mitochondrial damage and restores osteogenic differentiation by blocking the JNK signaling pathway.

Curculigoside is the primary active metabolite in the *Curculigo orchioides Gaertn.*, and has been demonstrated to possess multiple pharmacological effects, particularly anti-osteoporotic activity (Du et al., 2025). Research has found (ZHU et al., 2019) that Curculigoside protects OBs from dexamethasone-induced oxidative stress damage, significantly reduces ROS production, increases mitochondrial membrane potential levels, and promotes proliferation and differentiation of OBs. Additionally, it increases the relative ratio of osteoblast-inhibiting factors (such as OPG) to osteoclast-differentiating factors (e.g., RANKL). This effectively inhibits osteoclastogenesis while reducing inflammatory cytokine expression to suppress OC formation.

OP is frequently accompanied by oxidative stress. Research (Chen, L. et al., 2020a) indicates that Proanthocyanidins (PAC) can increase Nrf2 nuclear translocation and elevate downstream HOU-1 (heme oxygenase-1) expression levels. This helps restore MMP levels and reduce mtROS production while promoting the expression of bone formation-related genes such as Runx2, OCN, and COLI. PAC improves glucocorticoid-induced oxidative stress and mitochondrial dysfunction in OBs via Nrf2 activation, thereby protecting OB function and reducing the risk of OP. Similarly, another study (Zhang et al., 2014) confirmed PAC’s protective effects on mitochondrial function by restoring mitochondrial membrane potential levels and respiratory chain complex IV activity, reducing mtROS and mitochondrial superoxide, and improving H_2_O_2_-induced mitochondrial dysfunction. This inhibits OB apoptosis, suggesting PAC may suppress OP progression through its antioxidant and anti-apoptotic effects.

Similar to PAC, the natural flavonoid Kaempferol also protects OBs by restoring mitochondrial membrane potential levels and respiratory chain complex IV activity, thereby reducing mtROS production and maintaining Ca^2+^ homeostasis (Xie et al., 2021). Furthermore, Paeoniflorin, Astragalus polysaccharides, and Allicin can all improve OBs' survival and function by reducing ROS generation and maintaining mitochondrial function. Suh et al. (Kwang et al., 2013) demonstrated that Paeoniflorin, the primary active compound in *Paeonia lactiflora* and *Paeonia veitchii*, reduces ROS and reactive nitrogen species (RNS) levels, protects mitochondrial function, improves cellular viability, and decreases apoptosis of OBs. Astragalus polysaccharides, the primary active component in *Astragalus membranaceus (Fisch.) Bunge*, can inhibit mitochondrial ROS production, reduce BMSC apoptosis, and enhance BMSC proliferation and pluripotency (Yang et al., 2016). In addition, Gastrodin can inhibit ROS production and suppress apoptosis of OBs by activating the Nrf2 signaling pathway (Liu, S. et al., 2018).

Regulating oxidative stress is one of the most common mechanisms of action for CBD-derived natural chemical metabolites, though the modes of action and strength of evidence vary among different compounds. The mechanisms of NGR1 and PAC are relatively well understood, involving not only ROS scavenging but also restoration of mitochondrial membrane potential and ATP levels, activation of key antioxidant transcription factors (such as Nrf2), or inhibition of pro-apoptotic signaling (such as JNK) (Chen, L. et al., 2020a; Li et al., 2021). Notably, PAC has demonstrated reliable evidence of protecting mitochondrial complex IV activity in two independent studies (Zhang et al., 2014). While Kaempferol and Paeoniflorin also exhibit antioxidant and mitochondrial protective effects, their mechanisms remain relatively poorly understood, with research primarily focused on phenotypic observations (Kwang et al., 2013; Xie et al., 2021). Astragalus polysaccharide, as a macromolecular metabolite, requires further clarification of its direct cellular mechanisms (e.g., whether it acts via membrane receptors) (Yang et al., 2016). Overall, NGR1 and PAC hold greater developmental advantages in antioxidant strategies due to their clearer signaling pathway regulation and comprehensive protective effects on mitochondrial function.

The above studies clearly demonstrate that CBD-derived natural chemical metabolites hold potential for treating OP by regulating mitochondrial oxidative stress to improve OB function and enhance osteogenic differentiation of BMSCs. However, research on their inhibitory effects on OCs remains relatively scarce. Future efforts should focus on exploring the systematic mechanisms of CBD in bidirectionally regulating osteogenic differentiation and osteoclast differentiation, thereby advancing the development of multi-target synergistic intervention strategies.

#### 4.1.4 Natural chemical metabolites improve mitophagy to alleviate OP

Ferutinin is a natural non-steroidal phytoestrogen metabolite found in high concentrations in *Allium sativum L.* and *Ligusticum sinense 'Chuanxiong'*. Acting as an estrogen receptor agonist, it exhibits antioxidant and anti-free radical activity while playing a crucial role in regulating mitochondrial function. Maiti et al. (Maity et al., 2022) found that ferulic acid reduces levels of mitochondrial membrane potential, ATP, and ROS while increasing the expression of PINK1 and Parkin, thereby inducing mitophagy and promoting differentiation of OBs. Therefore, Ferutinin is a natural compound worth exploring in depth for its anti-OP properties.

Epigallocatechin-3-gallate (EGCG) is a natural botanical chemical metabolite extracted from *Camellia sinensis (L.) Kuntz*. Sarkar et al. (Sarkar et al., 2022) found that EGCG inhibits osteoclast differentiation by regulating mitophagy and mitochondrial function. EGCG dose-dependently reduced mtROS production and mitochondrial membrane potential while increasing ATP production *in vitro* and in primary bone marrow cells via the AKT and p38 MAPK signaling pathways. Concurrently, it promoted the expression of PINK1 and Parkin. By inhibiting mRNA and protein expression of mitophagy-related molecules, it reduces the expression of osteoclast differentiation markers, suppresses osteoclast differentiation in bone cells, mitigates bone loss, and consequently delays the progression of OP.

*Schisandra chinensis* is a commonly used CBD with effects including tonifying the kidneys and nourishing the lungs, as well as protecting the liver. Kim et al. (Kim et al., 2019) combined *S. chinensis* extract (SCE) with exercise and found it improved skeletal muscle regeneration in OVX rats, induced osteogenic differentiation, and regulated bone formation and resorption in OVX rats. Concurrently, SCE significantly suppressed inflammatory molecule expression and β-galactosidase activity in RAW264.7 and aged HDF cells. It enhanced mitochondrial biogenesis and mitophagy by increasing antioxidant activity through mtROS downregulation. Thus, SCE may represent a potential natural chemical metabolite for preventing OP.

Cistanoside A, a natural chemical metabolite extracted from *Cistanche deserticola Ma*, one of the most commonly used CBDs in clinical practice, has demonstrated significant anti-osteoporotic effects (Xiong et al., 1996; Xu et al., 2017). Chen T et al. (2022) found that Cistanoside A enhances osteogenic differentiation and mineralization by increasing intracellular LC3-I and enhancing Wnt/β-catenin signaling pathway activity. The addition of the autophagy inhibitor 3-MA inhibited osteogenic differentiation and suppressed Wnt/β-catenin signaling activity, increasing apoptosis while reducing mitophagy. The combination of Cistanoside A and DKK-1 resulted in higher levels of apoptosis but lower autophagy levels. Therefore, based on the Wnt/β-catenin signaling pathway, Cistanoside A acts as an effective inducer of mitophagy and inhibitor of apoptosis in primary OBs, thereby enhancing osteogenic differentiation.

Regulating mitophagy has been a research hotspot in recent years. Studies on EGCG are highly representative, not only confirming its promotion of mitophagy via the PINK1/Parkin pathway but also linking this mechanism for the first time to inhibiting osteoclast differentiation, offering new insights for developing anti-resorptive drugs (Sarkar et al., 2022). Its effective concentration (25–50 μM) also falls within the classical range. Ferutinin, while effective in promoting osteogenesis, simultaneously reduces mitochondrial membrane potential and ATP. This “mitochondrial damage-induced” approach to activating autophagy differs from the classical concept of “protective mitophagy,” necessitating vigilance regarding its long-term safety (Maity et al., 2022). Cistanoside A links mitophagy to the Wnt/β-catenin signaling pathway, providing a molecular basis for the “multi-target” regulation observed in CBD formulations (Chen T. et al., 2022). Future research in this field should focus on distinguishing between “survival-promoting mitophagy” and “autophagic cell death,” and clarifying the differential effects of these metabolites in OCs versus OBs.

### 4.2 CBD formulations for OP treatment by regulating mitochondrial dysfunction

Unlike CBD-derived natural chemical metabolites, CBD formulations exert therapeutic effects through multi-component, multi-target synergistic interactions. Based on the principle of “sovereign, minister, assistant, courier,” these formulations simultaneously modulate multiple processes related to mitochondrial dysfunction—including biogenesis, dynamics, oxidative stress, and mitophagy—thereby achieving holistic regulation of bone metabolism. However, due to the complexity of their chemical composition, the molecular mechanisms underlying their synergistic effects remain poorly understood, and systematic comparisons of their efficacy in OP models are lacking. This section summarizes current evidence on representative mitochondrial dysfunction-targeting CBD formulations and highlights key research gaps.

Estrogen deficiency is one of the primary causes of OP. Its mechanism involves PGC-1α and steroid receptor coactivator-3 (SREBP-3)—they influence bone metabolism by regulating estrogen receptors on osteocytes. Bushen Zhuanggu Granules (BSZGG) is a CBD formulation clinically used for OP and bone loss. Modern pharmacological research has also confirmed its ability to modulate bone metabolism through multiple targets and enhance bone density. Chen et al. (CHEN et al., 2019) observed elevated serum PGC-1α and SREBP-3 expression in OVX rats following BSZGG intervention. As both markers positively correlate with bone mineral density, they may serve as potential predictors of OP severity. In summary, one of the targets of BSZGG in treating OP is mitochondrial biogenesis.

Tenghuangjiangu Capsule (THJGC), primarily composed of CBDs, including *Rehmannia glutinosa (Gaetn.) Libosch. ex Fisch. et Mey., C. deserticola Ma*, and *E. brevicornu Maxim.*, possess effects of tonifying the kidneys and strengthening bones, promoting blood circulation, and alleviating pain. An et al. (AN et al., 2023) found that in OVX rats, SIRT1 expression was significantly reduced. THJGC intervention activated SIRT1, promoted PGC-1α expression, and subsequently elevated Nrf2 and Runx2, enhancing OB antioxidant stress resistance and increasing bone formation. This effect is presumed to be related to PGC-1α′s role in regulating mitochondrial biogenesis. Additionally, it was found (AN et al., 2024) that THJGC also influences the Bax/Bcl-2 ratio by activating the SIRT1/PGC-1α signaling pathway, thereby reducing caspase-3 and caspase-9 expression, effectively inhibiting apoptosis of OCs and reducing bone loss.

THJGC simultaneously regulates OB and OC functions through multi-component synergistic effects. Icariin in the formulation has been demonstrated to modulate mitochondrial dynamics (Yao et al., 2019), Cistanoside A activates mitophagy (Chen M et al., 2022), and Astragalus polysaccharides mitigate oxidative stress (Yang et al., 2016). These components may produce synergistic effects through distinct mitochondrial dysfunction pathways: on one hand, activating the SIRT1/PGC-1α/Nrf2 pathway to promote osteoblast survival; on the other hand, inhibiting osteoclast differentiation via the SIRT1/NF-κB/NLRP3 pathway. This “bidirectional regulation and synergistic enhancement” mechanism exemplifies the compound’s characteristic of integrated regulation through “multiple metabolites-multiple targets-multiple pathways.”

Zuo gui pill (ZGP) and You gui pill (YGP) target kidney yang and kidney yin deficiency, respectively, demonstrating significant efficacy in treating OP caused by kidney deficiency in postmenopausal women (Wang et al., 2023). Yao (Yao, 2022) administered ZGP and YGP to OVX rats, finding both reduced bone marrow fat volume—closely linked to activation of the PGC-1α signaling pathway—while also elevating UCP1 protein expression. In addition, YGP demonstrated a more pronounced effect in promoting brown adipose tissue’s regulation of lipids. Both formulations increased serum ATP and SOD production, clearing the large accumulation of ROS in mitochondria and thereby mitigating oxidative stress damage. Furthermore, ZGP and YGP promote Cyt C, cyclooxygenase-1 (COX-1), and ATP5α1 protein expression. This leads to enhanced enzymatic activity in mitochondrial respiratory chain complexes I and II, thereby positively regulating cellular mitochondrial biogenesis.

Comparing the functional characteristics of ZGP and YGP, both improve mitochondrial biogenesis by activating the PGC-1α signaling pathway, but their synergistic mechanisms differ: ZGP primarily enhances the activity of mitochondrial respiratory chain complexes I/II (Cyt C, COX-1, ATP5α1↑), directly enhancing energy metabolism. Beyond these effects, YGP more significantly activates UCP1 expression, suggesting it may indirectly regulate bone-fat balance by promoting brown fat thermogenesis (151). This divergence may stem from the distinct “nourishing yin” (ZGP) and “warming yang” (YGP) formulations: ZGP ingredients like *R. glutinosa (Gaetn.) Libosch. ex Fisch. et Mey.* and *Cornus officinalis Siebold and Zucc*—which nourish kidney yin—likely primarily target osteoblast mitochondria; whereas YGP components such as *Aconiti Lateralis Radix Praeparata* and *Cinnamomum cassia (L.) D. Don*—warming yang agents—may synergistically enhance osteogenic differentiation by regulating systemic energy metabolism (e.g., via brown adipose tissue activation). This synergistic mechanism of “formulation composition-multitarget regulation-organ interplay” represents the unique advantage of formulations over single-chemical metabolites.

Insulin resistance is one of the common characteristics in patients with OP (Petersen and Shulman, 2018). Research confirms that the expression of the key glucose transporter GLUT4 is reduced in the adipose tissue of OVX rats. The quantity of GLUT4 determines the level of glucose metabolism in skeletal muscle and adipocytes, suggesting impaired glucose tolerance and insulin resistance in the model rats. This may be closely related to the disruption of fat metabolism caused by estrogen deficiency. Erzhiwan (EZW), composed of *Ligustrum lucidum Ait*. and *Eclipta prostrata (Linn.) Linn*., nourishes the liver and kidneys while strengthening the lower back and knees (TIAN et al., 2020). Han et al. (HAN et al., 2022) observed that EZW intervention activated the SIRT1/PGC-1α/GLUT4 signaling pathway in adipose tissue of OVX rats, effectively increasing glucose uptake and mitochondrial function in adipocytes, thereby improving glucose metabolism disorders in adipose tissue. Furthermore, as a clinically validated formulation for treating OP, the Bushen tongluo formula (BSTLF) was found (MIN et al., 2018) to increase bone density in OVX rats and reduce blood glucose levels in skeletal muscle adipocytes. Its mechanism may involve promoting GLUT4 expression by activating PGC-1α.

In summary, CBD modulates mitochondrial dysfunction through multi-target regulation (including but not limited to core mechanisms such as mitochondrial biogenesis, mitochondrial dynamics, mitochondrial oxidative stress, and mitophagy). It intervenes in the OP process by eliminating damaged mitochondria, regulating energy metabolism, and balancing osteogenic-osteoclastic differentiation. In-depth research has elucidated the significant clinical value of CBD in treating OP, enhancing scientific recognition and acceptance of CBD for OP prevention and treatment. This provides crucial theoretical support for developing mitochondrial-targeted therapeutic strategies for bone metabolic diseases. Detailed experimental information on relevant CBD-derived natural chemical metabolites and formulations is presented in Tables 2, 3.

**TABLE 2:** Natural chemical metabolites for OP treatment by regulating mitochondrial dysfunction.

Targets	Natural chemical metabolites	Resource	*In Vivo*/*In Vitro*	Models	Dose	Duration time	Negative/positive control (NC/PC)	Statistical significance	Outcomes	Mechanism	Potential limitations/controversies	References
Mitochondrial biogenesis	Resveratrol	*Polygonum cuspidatum Sieb.et Zucc*	*In Vitro*	PO-MSCs	500 nM and 5 μM	5 and 10 days; 2 and 3 weeks	NC: ; PC:	*P < 0.05 (5 μM group)	Promoted osteogenic differentiation of PO-MSCs	ALP, mtDNA, mitochondrial mass↑	Low bioavailability	Moon et al. (2020)
​	Anthocyanins	*Vitis vinifera L*	*In Vivo/In Vitro*	hFOB1.19 Medaka	1.0 μg/mL 5 μg/mL	72 h 5 days	NC: ; PC: resveratrol (Activated the SIRT1/PGC-1α pathway)	**P < 0.01	Promoted osteogenic differentiation and reduced bone resorption	SIRT1/3 and PGC-1α ↑; HDAC1, p53, and Bax/Bcl-2 ↓	The activity of natural chemical metabolites remains unclear, with significant variations across species	Ren et al. (2021)
​	Liquiritigenin	*Glycyrrhiza uralensis Fisch*	*In Vitro*	MC3T3-E1 cells	0.01, 0.1, and 1 μM	48 h	NC: ; PC:	*P < 0.05 (1 μM group)	Inhibited apoptosis of OBs and promoted osteogenic differentiation	MMP, SOD, ATP, Complex IV, and PGC-1α ↑; ROS ↓	*In vitro* studies only; no *in vivo* efficacy data available	Suh et al. (2014)
​	Genistein	*Glycine* max *(L.) Merr*	*In Vitro*	Sham-BMMSCs and OVX-BMMSCs	10^−3^, 10^−2^, 10^−1^, 1, and 10 μM	24, 48, and 72 h	NC: ; PC:	***P < 0.001 (10 μM group)	Reduced the senescence of BMSCs	ERRα and PGC-1α ↑; ROS ↓	Estrogen-like effects require attention	Li, M. et al. (2023)
​	1,2,3,4,6-Pentagalloylglucose (PGG)	*Schisandra chinensis (Turcz.) Baill. and Paeonia lactiflora Pall*	*In Vivo/In Vitro*	MC3T3-E1 cells and BMSCs Zebrafish	10^−10^, 10^−9^, and 10^−8^	14 days; 4 days	NC: PC: 17 β-estradiol (10^−8^ M) (Activated the ERRα/PGC-1α pathway)	**P < 0.01	Inhibited the apoptosis of OBs	Nrf2↑; ROS↓; Activated the Nrf2/HO-1 signaling pathway	Extremely low concentrations (pM-nM), with physiological significance questionable; mammalian data lacking	CHEN, T.-t. et al. (2020)
​	Morinda officinalis polysaccharide	*Morinda officinalis*	*In Vivo*	SD rat (OVX)	300 mg/kg	8 weeks	NC: DMSO (oral gavage) PC: ZLN005 (15 mg/kg) (oral gavage) (Activated the PGC-1α/ERRα pathway)	***P < 0.001	Enhanced the antioxidant capacity and improved OP in OVX rats	SOD, GSH, GSH-Px ↑; MDA ↓; Inhibited the PGC-1α/PPARγ signaling pathway	Mechanistic contradictions (inhibition of PGC-1α) run counter to prevailing understanding and warrant cautious interpretation	Rong et al. (2022)
​	Gastrodin	*Gastrodia elata Blume*	*In Vitro*	Primary OBs	1.5 μM	10 days	NC: ; PC:	*P < 0.05 (5 μ M group)	Promoted osteogenic differentiation and the formation of osteogenic nodules	Nrf2 ↑; Activated the Nrf2/Keap1 signaling pathway	*In vitro* studies only; no *in vivo* data available	YIN et al. (2020)
Mitochondrial dynamics	Curcumin	*Curcuma longa L*	*In Vitro*	Saos-2	10, 20, 30, 40, and 50 μM)	24 h	NC: ; PC:	***P < 0.001 (30 μM group)	Inhibited apoptosis of OBs, promoted differentiation of OCs	Drp1 and mTOR ↓; Activated the Akt-GSK3β signaling pathway	Extremely low bioavailability	Dai et al. (2017)
​	Icariin	*Epimedium brevicornu Maxim*	*In Vitro*	BMSCs	0.01, 0.1 1, and 10 mM	48 h	NC: ; PC:	**P < 0.01 (1 mM group)	Inhibited apoptosis of BMSCs, promoted their osteogenic differentiation and proliferation	FIS1 and MFN2 ↑; DRP1/Cyt C↓; Activated the PI3K/AKT/mTOR signaling pathway; Inhibited the ERK/JNK signaling pathway	Excessively high concentrations (at the mM level) raise questions about their physiological significance	Yao et al. (2019)
​	Silibinin	*Silybum marianum (L.) Gaertn*	*In Vitro*	MC3T3-E1 cells	100 μM	Not mentioned	NC: ; PC: MitoQ (1 μM), CsA (1 μM), and FPS-ZM1 (40 μM) (MitoQ: Scavenge mtROS, protected mitochondrial membrane potential; CsA: Inhibited mPTP opening, reduced mitochondrial apoptosis; FPS-ZM1: Inhibited RAGE, reduces AGEs-induced mitochondrial damage)	**P < 0.01	Inhibited apoptosis of OBs	L-Opa1 ↑; S-Opa and Fis1 ↓; Downregulated the AGEs-RAGE signaling pathway	Numerous positive controls indicate that self-specificity requires further validation. Positive controls are numerous, and their specificity requires further validation	Mao et al. (2018)
Mitochondrial oxidative stress	Notoginsenoside R1	*Panax notoginseng (Burkill) F. H. Chen ex C. H. Chow*	*In Vitro*	MC3T3-E1 cells	10, 25, or 50 μM	4 weeks	NC: PC:	***P < 0.001 (50 μM group)	Promoted osteogenic differentiation	MMP, ATP, and mtDNA copy number ↑; ROS ↓; Inhibited the JNK signaling pathway	*In vitro* studies only; no *in vivo* efficacy validation	Li et al. (2021)
​	Curculigoside	*Curculigo orchioides Gaertn*	*In Vitro*	OBs	25, 50, and 100 μg/mL	24 h	NC: ; PC:	**P < 0.01 (100 μg/mL group)	Promoted proliferation and differentiation of OBs	MMP, ALP, OPG, BMP-2, β-catenin, IGF-1, and M-CSF ↑; RANKL, RANK, and ROS ↓	Effective concentration is expressed in μg/mL; molar concentration is unknown	ZHU et al. (2019)
​	Proanthocyanidins (PAC)	*Vitis vinifera L*	*In Vitro*	MC3T3-E1 cells	0.01, 0.1, and 1 μM	6, 12, and 24 h	NC: ; PC:	*P < 0.05 (1 μ group)	Improved mitochondrial function of OBs; inhibited apoptosis of OBs	Nrf2 and MMP ↑; mTOR ↓; Inhibited the p53 signaling pathway	Complex composition, active metabolites not yet determined	Chen, L. et al. (2020a), Zhang et al. (2014)
​	Kaempferol	*Kaempferia galanga L*	*In Vitro*	MC3T3-E1 cells	1, 5, 10, 25, 50, 75, and 100 μM	14 days	NC: ; PC:	**P < 0.01	Alleviated the inhibitory effect of DEX on osteogenic differentiation	Cyclin D1 and Bcl-2 ↑; Activated JNK and p38-MAPK signaling pathways	Wide range of effective concentrations, with unclear specific targets Effective concentration range is broad, with unclear specific targets	Xie et al. (2021)
​	Paeoniflorin	*Paeonia lactiflora Pall*	*In Vitro*	MC3T3-E1 cells	0.01, 0.1, and 1 μM	14 days	NC: ; PC: antimycin A (Inhibited mitochondrial complex III, induced mtROS production and mitochondrial apoptosis)	*P < 0.05	Inhibited oxidative damage of OBs, improved mitochondrial function, and reduced apoptosis of OBs	Cyt C↑; ROS ↓	Research on the mechanism remains superficial, primarily confined to phenotypic observations	Kwang et al. (2013)
​	Astragalus polysaccharide	*Astragalus membranaceus (Fisch.) Bunge*	*In Vitro*	BMSCs	10, 30, 100, and 300 μg/ml	24 h	NC: ; PC:	***P < 0.001 (300 μg/mL group)	Inhibited the senescence and apoptosis of BMSCs, promoted their osteogenic differentiation	mtROS↓	Macromolecules, direct mechanism of action unknown	Yang et al. (2016)
​	Gastrodin	*Gastrodia elata Blume*	*In Vivo/In Vitro*	OBs GIO rat	1 or 5 μM; 1 and 5 mg/kg/day	7 days; 8 consecutive weeks	NC: ; PC:	**P < 0.01 (5 mg/kg/d group)	Inhibited apoptosis of OBs	ROS ↓; Activated Nrf2 signaling pathway	Dose-dependent studies remain insufficient; Dose-dependent studies are not yet sufficient	Liu, S. et al. (2018)
​	Luteolin	*Chrysanthemum morifolium Ramat*	*In Vivo/In Vitro*	MC3T3-E1 OVX mice	0.04, 0.2, 0.5, 1, and 2 μM 1 and 20 mg/kg/day	24, 48, 72, and 96 h 12 weeks	NC: CMC-Na (oral gavage); PC: E2 (0.104 mg/kg) (oral gavage) (Inhibited the RANKL/OPG pathway)	***P < 0.001	Inhibited apoptosis of OBs	MMP ↑; mitochondrial Cyt c, ROS ↓; Activated the PI3K-AKT signaling pathway	The mechanism is comprehensive, but the core target still needs to be identified	Chai et al. (2024)
​	Honokiol	*Houpoea officinalis (Rehder and E. H. Wilson) N. H. Xia and C. Y. Wu*	*In* *Vitro*	MC3T3-E1 cells	0.01, 0.1, and 1 μM	14 days	NC: ; PC:	*P < 0.05	Improved mitochondrial function and protected OBs from damage	MMP, SOD, and Complex IV ↑; ROS ↓; Regulated the PI3K/CERE signaling pathway	*In vitro* studies only; no *in vivo* validation	Choi (2011)
Mitophagy	Ferutinin	*Allium sativum L. and Ligusticum sinense 'Chuanxiong'*	*In Vitro*	Dental pulp-derived stem cell (DPSC)	-	12, 24, and 48 h	NC: ; PC:	**P < 0.01	Promote the differentiation of OBs	PINK1, Parkin↑; ATP, MMP, mtROS↓	Potential safety concern (induction of mitochondrial damage to initiate mitophagy)	Maity et al. (2022)
​	Epigallocatechin-3-gallate	*Camellia sinensis (L.) Kuntze*	*In Vitro*	OCs	5, 10, 25, 50 and 100 µM	24 and 48 h	NC: ; PC:	***P < 0.001	Inhibited the differentiation of OCs	PINK1, Parkin, ATP ↑; mTOR ↓; Activated the AKT and p38 MAPK signaling pathways	Concentration-dependent bidirectional effects require attention	Sarkar et al. (2022)
​	Schisandra chinensis extract (SCE)	*Schisandra chinensis (Turcz.) Baill*	*In Vivo/In Vitro*	RAW 264.7 cells; HDF cells, C2C12 cells, and MC-3T3 E1 cells; BMMs; OVX rats	1, 5, 10, 20, 30, 40 μg/mL; 10 mg/kg/day	12 days 8 weeks (5 days a week)	NC: CMC-Na PC: exercise	**P < 0.01	Promoted mitochondrial biogenesis and autophagy, induced osteogenic differentiation, and regulated bone formation and resorption processes in OVX rats	Inflammatory molecules, β-galactosidase, mtROS ↓	Crude extract, active metabolites unknown	Kim et al. (2019)
​	Cistanoside A	*Cistanche deserticola Ma*	*In Vitro*	OBs	5, 10, 20, 40, 80, and 160 μM	7 days; 2 weeks	NC: ; PC:	***P < 0.001	Reduced apoptosis and promoted autophagy	LC3-I/II ↑ Activated the Wnt/β-catenin signaling pathway	Wide concentration range; optimal concentration requires optimization	Chen T. et al. (2022)

**TABLE 3:** CBD formulations for OP treatment by regulating mitochondrial dysfunction.

CBD formulations	Formulation composition	*In Vivo*/In Vitro	Models	Dose	Duration time	Negative/positive control (NC/PC)	Statistical significance	Outcomes	Mechanism	References
Zuigui Pill (ZGP)	*Rehmannia glutinosa (Gaetn.) Libosch. ex Fisch. et Mey., Cornus officinalis Siebold and Zucc., Dioscorea opposita Thunb., Cuscutachinensis Lam., Lycium chinense Miller, and Achyranthes bidentata Blumeetc.*	*In Vivo*	Postmenopausal Osteoporosis (PMOP)rats	9.69 g/kg/d (oral gavage)	12 weeks	NC: PC: Estradiol Valerate Tablets (0.09 mg/kg/day)	***P < 0.001	Enhanced the osteogenic differentiation capacity of rat bone marrow tissue	PGC-1α, NRF1/2, TFAM ↑	Yao (2022)
Yougui Pill (ZGP)	*Rehmannia glutinosa (Gaetn.) Libosch. ex Fisch. et Mey., Cornus officinalis Siebold and Zucc., Dioscorea opposita Thunb., Angelica sinensis, Eucommia ulmoides Oliv., Aconitum carmichaeli Debx., and Cinnamomum cassia (L.) D. Donetc.*	*In Vivo*	PMOP rats	10.52 g/kg/d (oral gavage)	12 weeks	NC: PC: Estradiol Valerate Tablets (0.09 mg/kg/day) (oral gavage)	***P < 0.001	Enhanced mitochondrial respiratory function, antioxidant capacity, and osteogenic differentiation potential in bone marrow tissue	PGC-1α, NRF1/2, and TFAM↑	Yao (2022)
Tenghuangjiangu capsule (THJGC)	*Rehmannia glutinosa (Gaetn.) Libosch. ex Fisch. et Mey., Davallia trichomanoides Blume, Cistanche deserticola Ma, Epimedium brevicornu Maxim., and Spatholobus suberectus Dunnetc.*	*In Vivo*	PMOP rats	0.09, 0.18, and 0.36 g/kg/d (oral gavage)	2 months	NC: PC: Estradiol Valerate Tablets (0.09 mg/kg/day) (oral gavage)	***P < 0.001 (0.36 g/kg/d group)	Inhibited apoptosis and promoted osteogenesis of OBs	Bcl-2 ↑ Caspase-3 and Caspase-9 ↓; Promoted the SIRT1/PGC-1α/Nrf2 signaling pathway	AN et al. (2024)
Erzhiwan (EZW)	*Ligustrum lucidum Ait. and Eclipta prostrata (Linn.) Linn*	*In Vivo*	Osteoporotic rats	1.6 g/kg/d (oral gavage)	8 weeks	NC: PC: Alendronate sodium (1 mg/kg/day) (oral gavage)	**P < 0.01	Improved glucose metabolic disorders in adipose tissue under postmenopausal OP conditions	GLUT4 ↑ Promoted the SIRT1/PGC-1α/GLUT4 signaling pathway	HAN et al. (2022)
Bushen Tongluo Decoction (BSTLD)	*Epimedium brevicornu Maxim., Davallia trichomanoides Blume, Poria cocos(Schw.)Wolf, Cynanchum otophyllum Schneid., and Glycyrrhiza uralensis Fischetc.*	*In Vivo*	Osteoporotic rats	6.3 g/kg/d (oral gavage)	70 days	NC: PC:	**P < 0.01	Enhanced glucose metabolism	PGC-1α and GLUT4 ↑	MIN et al. (2018)
Bushen Zhuanggu Granules (BSZGG)	*Davallia trichomanoides Blume, Pleuropterus multiflorus (Thunb.) Turcz. ex Nakai, Poria cocos(Schw.)Wolf, Cynanchum otophyllum Schneid., Angelica sinensis, Codonopsis pilosula (Franch.) Nannf., Rehmannia glutinosa (Gaetn.) Libosch. ex Fisch. et Mey., and Polygonatum sibiricum Delar. ex Redouteetc.*	*In Vivo*	OVX rats	2.5 m g/kg/d	12 weeks	NC: PC: Alendronate sodium (7 mg/kg/day) (oral gavage)	***P < 0.001	Promoted bone formation and increased bone density	PGC-1α and SRC-3 ↑	CHEN et al. (2019)
Lurong Jiangu Capsules	*Cervi Cornu Pantotrichum, Pleuropterus multiflorus (Thunb.) Turcz. ex Nakai, Eucommia ulmoides Oliv., Angelica sinensis, and Panax notoginseng (Burkill) F. H. Chen ex C. H. Chowetc.*	*-*	Patients with OP	0.36 g/tablet (5 tablets/dose, 3 doses/d)	6 months	NC: ; PC: 0.25 μg/dose, 3 doses/day	*P < 0.05	Increased bone mineral density and improved bone metabolism indicators	Serum human bone alkaline phosphatase (BALP) and type I collagen carboxy-terminal peptide (CTX) ↓	MA et al. (2019)

To further extend these findings to clinical practice, Table 4 summarizes the most commonly used CBD formulations in the clinical management of osteoporosis, providing detailed information on their composition, indications, standard clinical dosage, course of medication, common combination regimens with conventional anti-osteoporotic drugs, and potential interactions and synergistic effects. This table serves as a practical reference for clinicians and offers a foundation for future translational research exploring the integrative use of CBDs and Western medicines in OP therapy.

**TABLE 4:** Current CBD formulations for treating OP in clinical practice.

Formulations	Formulation composition	Types of osteoporosis	Standard clinical dosage	Course of medication	Combination therapy regimens	Interaction and combination effects	References
Zuogui Pill (ZGP)	*Rehmannia glutinosa (Gaetn.) Libosch. ex Fisch. et Mey., Cornus officinalis Siebold and Zucc., Dioscorea opposita Thunb., Cuscutachinensis Lam., Lycium chinense Miller, and Achyranthes bidentata Blumeetc.*	Postmenopausal osteoporosis, Senile osteoporosis	Water-honeyed pills: 9 g per dose, twice daily, orally Large honey pills: 1 pill per dose, twice daily, orally	A treatment course lasts 3–6 months and may be extended based on the patient’s condition	① Calcium supplements (calcium carbonate, calcium citrate) + Vitamin D ② Bisphosphonates (alendronate sodium 70 mg/week) ③ Selective estrogen receptor modulators (raloxifene 60 mg/day)	Combination effects: Synergistically increases bone density, alleviates symptoms of kidney yin deficiency such as lower back and knee weakness, dizziness, and tinnitus, while reducing side effects Interactions: When combined with oral bisphosphonates, take at least 2 h apart (Zuo Gui Wan contains mineral components that may affect bisphosphonate absorption). Concurrent use with raloxifene may produce synergistic estrogen-like effects; endometrial thickness requires monitoring (theoretical hypothesis; clinical studies insufficient)	Han (2019)
Yougui Pill (YGP)	*Rehmannia glutinosa (Gaetn.) Libosch. ex Fisch. et Mey., Cornus officinalis Siebold and Zucc., Dioscorea opposita Thunb., Angelica sinensis, Eucommia ulmoides Oliv., Aconitum carmichaeli Debx., and Cinnamomum cassia (L.) D. Donetc.*	Postmenopausal osteoporosis, senile osteoporosis, and glucocorticoid-induced osteoporosis	Water-honeyed pills: 9 g per dose, twice daily, orally Large honey pills: 1 pill per dose, twice daily, orally	A treatment course lasts 3–6 months	① Calcium supplements + Vitamin D ② Bisphosphonates ③ Teriparatide (20 μg daily subcutaneous injection) ④ Calcitonin (Salmon calcitonin nasal spray)	Combination effects: Enhances bone formation and alleviates symptoms of kidney yang deficiency, such as cold sensitivity, cold limbs, and cold pain in the lower back and knees; may synergistically promote bone formation when used with teriparatide Interactions: Contains *Aconitum carmichaeli Debx*., whose alkaloid components may interact with drugs prolonging the QT interval (e.g., certain antiarrhythmics, quinolone antibiotics); Contains *Cinnamon,* INR monitoring is required when combined with anticoagulants (Warfarin) (theoretical risk)	Cao et al. (2018)
Tenghuang Jiangu Capsule/Tablet (THJGC/THJGT)	*Rehmannia glutinosa (Gaetn.) Libosch. ex Fisch. et Mey., Davallia trichomanoides Blume, Cistanche deserticola Ma, Epimedium brevicornu Maxim., and Spatholobus suberectus Dunnetc.*	Postmenopausal osteoporosis, senile osteoporosis with osteoarthritis, or significant osteoporotic pain	Capsules: 3 to 4 capsules per dose (0.5 g per capsule), three times daily, orally Tablets: 3 to 4 tablets per dose, three times daily, orally	A treatment course lasts 3–6 months	① Calcium supplements + Vitamin D ② Bisphosphonates ③ NSAIDs (e.g., celecoxib, for pain relief) ④ Chondroprotectants (glucosamine)	Combination effects: Improves bone density, significantly alleviates bone pain, and delays the progression of osteoarthritis Interactions: Contains *Salvia miltiorrhiza Bunge*. Concurrent use with anticoagulants (warfarin) may enhance anticoagulant effects and increase bleeding risk; INR monitoring is required. Concurrent use with antiplatelet agents (aspirin, clopidogrel) requires vigilance for bleeding tendencies. Concurrent use with NSAIDs may provide synergistic analgesic effects, but gastrointestinal protection is necessary	FENG et al. (2018)
Erzhiwan (EZW)	*Ligustrum lucidum Ait. and Eclipta prostrata (Linn.) Linn*	Postmenopausal osteoporosis (early stage), Bone mass reduction	Water-honeyed pills: 9 g per dose, twice daily, orally Concentrated Pills: 20 pills per dose, twice daily, orally	A 3-month period constitutes one treatment course, which may be taken consecutively	① Calcium supplements + Vitamin D ② Bisphosphonates ③ Combination with hypoglycemic agents for diabetic patients (metformin, sulfonylureas, etc.)	Combination effects: Improves bone metabolism markers (reduces bone resorption markers), offering dual benefits for diabetic patients with osteoporosis (enhances insulin sensitivity) Interactions: *Ligustrum lucidum Ait. and Eclipta prostrata (Linn.) Linn.* may enhance metformin’s hypoglycemic effect by modulating the AMPK pathway. When combined, blood glucose should be monitored appropriately to prevent hypoglycemia. Theoretically, synergistic hypoglycemic effects may occur when used with other antidiabetic medications	LIU et al. (2025)
Bushen Zhuanggu Granules (BSZGG)	*Davallia trichomanoides Blume, Pleuropterus multiflorus (Thunb.) Turcz. ex Nakai, Poria cocos(Schw.)Wolf, Cynanchum otophyllum Schneid., Angelica sinensis, Codonopsis pilosula (Franch.) Nannf., Rehmannia glutinosa (Gaetn.) Libosch. ex Fisch. et Mey., and Polygonatum sibiricum Delar. ex Redouteetc.*	Postmenopausal osteoporosis, Senile osteoporosis	10 g per dose, twice daily, taken with warm water	6 months	① Calcium supplements + Vitamin D ② Bisphosphonates ③ Active Vitamin D (Calcitriol)	Combination effects: Significantly increases bone density, improves bone metabolism indicators, and enhances the bone-protective effects of Western medications Interactions: Contains *Salvia miltiorrhiza Bunge*, requiring INR monitoring when combined with anticoagulants. Contains *Psoralea corylifolia Linn.*, whose furanocoumarin components may affect CYP450 enzyme activity. Exercise caution when co-administered with hepatically metabolized drugs (e.g., statins, certain calcium channel blockers), though no clinical interactions have been reported to date	DU et al. (2016)
Lurong Jiangu Capsules (LRJGC)	*Cervi Cornu Pantotrichum, Pleuropterus multiflorus (Thunb.) Turcz. ex Nakai, Eucommia ulmoides Oliv., Angelica sinensis, and Panax notoginseng (Burkill) F. H. Chen ex C. H. Chowetc.*	Severe osteoporosis, post-fracture healing phase, senile osteoporosis with sarcopenia	3 capsules per dose (0.4 g per capsule), three times daily, orally	6 months	① Calcium supplements + Vitamin D ② Bisphosphonates ③ Teriparatide ④ Anticoagulants/Antiplatelet agents (use with caution)	Combination effects: Promotes bone formation, accelerates fracture healing, and improves muscle strength and function Interactions: Contains *Panax notoginseng* (Burkill) F. H. Chen ex C. H. Chow, which exhibits antiplatelet effects. Concurrent use with aspirin, clopidogrel, warfarin, etc., may increase bleeding risk and requires close monitoring (especially in surgical patients). Contains deer antler, which has warming and tonifying properties. Hypertensive patients using it alongside antihypertensive medications should have their blood pressure monitored	Zhao X et al. (2024)
Bushen Tongluo Decoction (BSTLD)	*Epimedium brevicornu Maxim., Davallia trichomanoides Blume, Poria cocos(Schw.)Wolf, Cynanchum otophyllum Schneid., and Glycyrrhiza uralensis Fischetc.*	Osteoporosis with Chronic Bone Pain, Diabetic Osteoporosis	Decoction: One dose daily, boiled in water and divided into two servings Granules: Take as directed for 3 months	3 months	① Calcium supplements + Vitamin D ② Bisphosphonates ③ Antidiabetic drugs (metformin, insulin, etc.)	Combination Effects: Improves bone density, alleviates bone pain, and enhances glucose metabolism and insulin resistance in diabetic patients Interactions: Contains Astragalus membranaceus (Fisch.) Bunge, which may enhance immunomodulatory effects; caution is advised when combined with immunosuppressants (e.g., cyclosporine, tacrolimus). Contains *Salvia miltiorrhiza* Bunge, requiring INR monitoring when used with anticoagulants. May increase insulin sensitivity, necessitating blood glucose monitoring when combined with hypoglycemic agents	LUO et al. (2024)
Qianggu Capsules (QGC)	*Epimedium brevicornu Maxim., Morinda officinalis How, Davallia trichomanoides Blume, Astragalus membranaceus (Fisch.) Bunge, and Salvia miltiorrhiza Bungeetc.*	Primary osteoporosis, secondary osteoporosis (glucocorticoid-induced)	1 capsule per dose (0.25 g), three times daily, orally	3–6 months	① Calcium supplements + Vitamin D ② Bisphosphonates	Combination effects: Counteracts glucocorticoid-induced bone loss and reduces the risk of osteoporotic fractures Interactions: The primary active ingredient, total flavonoids of *Davallia trichomanoides Blume*, partially counteracts the bone resorption side effects of glucocorticoids without affecting their efficacy. When combined with bisphosphonates, it exhibits synergistic inhibition of bone resorption	PENG (2023)
Jintiange Capsules (JTGC)	*Rengong Hugu Fen*	Primary osteoporosis, post-fracture recovery phase	3 tablets per dose, 3 times daily, orally	3–6 months	① Calcium supplements + Vitamin D ② Bisphosphonates ③ Calcitonin	Combination effects: Alleviates bone pain, increases bone density, and promotes fracture healing Interactions: When used with bisphosphonates, take at least 2 h apart to avoid affecting bisphosphonate absorption; monitor total calcium intake when used with other calcium-containing medications	LIU, Z.-y. et al. (2018)
Gushukang Capsules/Granules (GSKC/GSKG))	*Epimedium brevicornu Maxim., Astragalus membranaceus (Fisch.) Bunge, Salvia miltiorrhiza Bunge, Rehmannia glutinosa (Gaetn.) Libosch. ex Fisch. et Mey., and Davallia trichomanoides Blumeetc.*	Primary osteoporosis, osteoporotic fractures	Capsules: 4 capsules per dose, twice daily, taken orally Granules: 10 g per dose, twice daily, dissolved in warm water	6 months	① Calcium supplements + Vitamin D ② Bisphosphonates ③ Estrogens or selective estrogen receptor modulators (raloxifene)	Combined effects: Synergistically increases bone density and alleviates symptoms such as lower back pain and fatigue Interactions: Contains blood-activating and stasis-resolving herbs like *Salvia miltiorrhiza Bunge* and *Ligusticum sinense 'Chuanxiong'.* Monitoring for bleeding risk is required when used with anticoagulants. Concurrent use with estrogen-based medications may increase the risk of endometrial hyperplasia (theoretical hypothesis; further research needed)	ZHOU et al. (2020)
Xianling Gubao Capsules (XLGBG)	*Psoralea corylifolia Linn., Epimedium brevicornu Maxim., and Salvia miltiorrhiza Bungeetc.*	Osteoporosis, fractures, arthritis, and avascular necrosis of the femoral head	3 tablets per dose, twice daily	3–6 months	① Calcium supplements + Vitamin D ②Calcium supplements + Vitamin D + Bisphosphonates	Combined effects: increases lumbar spine/hip bone mineral density and alleviates bone pain Interactions: No significant interactions identified	ZHANG et al. (2020)

## 5 Conclusions and perspectives

OP, a metabolic bone disease characterized by reduced bone mass and microstructural deterioration, arises from the disruption of the intricate multisystem, multi-pathway regulation governing bone remodeling (Lee et al., 2017). During bone remodeling, the dynamic equilibrium between OB-mediated bone formation and osteoclast-dominated bone resorption is compromised, leading to bone loss and microstructural degradation. Activation of signaling pathways such as Wnt/β-catenin and PI3K/AKT, or inhibition of the Notch pathway, promotes differentiation of BMSCs and OBs, while activation of the target of rapamycin complex 1 (TORC) promotes apoptosis of OCs (Liu et al., 2024). Furthermore, endocrine alterations, including declining levels of sex hormones, further exacerbate this imbalance. Current pharmacological interventions for OP primarily focus on two approaches: “anti-resorptive” and “pro-anabolic.” Anti-resorptive drugs, such as bisphosphonates and RANKL inhibitors, effectively slow bone loss by suppressing OC activity. Pro-anabolic agents, including parathyroid hormone analogues and anti-sclerostin antibodies, directly stimulate OB activity to enhance bone formation. Furthermore, foundational nutritional supplements like calcium and vitamin D remain crucial components of comprehensive management strategies. While effective in reducing fracture risk, their long-term safety and tolerability require further evaluation. Additionally, stem cell therapy, as an emerging treatment modality, demonstrates significant potential in bone regeneration and repair. However, numerous challenges persist in the practical application of stem cell therapy. Uncertainties regarding the post-transplantation fate of cells and their safety in recipients significantly constrain the advancement of human clinical trials (Jiang et al., 2020). In summary, constrained by the difficult-to-overcome issues of long-term safety and tolerability associated with OP medications, there is an urgent need to identify safe and effective therapeutic targets and strategies.

Mitochondria serve not only as organelles for energy metabolism but also as hubs for biological information transfer. Consequently, any impairment of mitochondrial function may disrupt mtDNA replication, energy production, and other critical processes, potentially leading to the development of related diseases. Recent studies (Li Z. et al., 2024) have revealed a close association between Recent studies have revealed a close association between mitochondrial dysfunction and mitochondrial metabolism and adaptive adjustments in OP. Under pathological conditions, factors such as mitochondrial oxidative stress, imbalance in fusion and fission, impaired mitophagy, and mtDNA mutations may all trigger mitochondrial dysfunction and the onset of OP. However, restoring mitochondrial homeostasis can mitigate oxidative damage caused by ROS and replenish mitochondrial reserves via biogenesis. Concurrently, it modulates mitochondrial shape and size through dynamics and maintains function by clearing damaged internal structures via mitophagy. Mechanistically, research on mitochondrial dysfunction regulation primarily focuses on targets such as DRP1, OPA1, GSK-3β, mTOR, SIRT1/3, PGC-1α, Nrf1/2, HIF-1α, PINK1, and Parkin. These pathways mediate the correction of mitochondrial dysfunction to regulate processes including activity, proliferation, differentiation, apoptosis, and senescence in osteogenesis-related cells such as BMSCs, OBs, and OCs. Therefore, mechanisms targeting mitochondrial dysfunction may represent potential therapeutic interventions for OP.

In recent years, CBD has provided new perspectives for the prevention and treatment of OP by regulating bone metabolic balance through targeted amelioration of mitochondrial dysfunction. CBD classifies OP under categories such as “bone atrophy,” “bone obstruction,” and “bone withering,” primarily attributing it to chronic systemic degenerative diseases caused by kidney essence deficiency, bone withering leading to marrow depletion, and loss of bone nourishment (ZHU et al., 2026). The guiding principle is “differential diagnosis and treatment, integrating disease and syndrome, holistic regulation, and combining prevention with treatment.” CBD therapies improve pathological states through multi-target, multi-step, and multi-level pathways, demonstrating significant promise and exploratory potential in preventing and treating OP. Regarding therapeutic stability, CBD typically focuses on regulating overall balance, aiming to modulate the immune system and enhance the body’s overall condition. For individualized treatment, CBD emphasizes syndrome differentiation and treatment. OP is typically categorized into syndromes such as kidney yang deficiency, liver-kidney yin deficiency, spleen-kidney yang deficiency, kidney deficiency with blood stasis, spleen-stomach deficiency, and blood stasis with qi stagnation. Treatment protocols must integrate patient constitution and disease characteristics to enhance specificity and efficacy. For instance, kidney yang deficiency may be addressed with the formulation You gui pill (Yao, 2022). CBD exerts effects through multiple targets, thereby enhancing therapeutic outcomes. Their metabolites, such as anthocyanins, act on both the SIRT1/3 and PGC-1 pathways as well as the Bax/Bcl-2 pathway. This promotes mitochondrial biogenesis and reduces mitochondrial apoptosis, multidimensionally improving pathological symptoms of OP (Chen, L. et al., 2020a; Zhang et al., 2014). Regarding safety, CBD generally exhibits superior safety and tolerability compared to certain pharmaceutical therapies. For instance, bisphosphonates have been observed to increase atypical femoral fractures, limiting their application in OP treatment (Black et al., 2020). Conversely, adverse reactions to CBD for OP are rarely reported, making it suitable for long-term disease management.

Although CBD holds certain advantages in regulating mitochondrial dysfunction to prevent OP, numerous challenges remain to be addressed: ① Despite the identification of multiple signaling pathways involved in ameliorating mitochondrial dysfunction, precise regulatory mechanisms require further investigation to discover specific and effective drug targets; ② Establishing specific regulatory networks to influence the metabolism of OBs and OCs remains a challenge and will be a key focus in further studies on the pathogenesis of OP; ③ Current research on CBD regulation of mitochondrial dysfunction to prevent OP is still limited to cellular and animal model experiments. High-quality, multicenter clinical trials are needed to determine its safety, efficacy, and scope of application; ④ Most current therapy research targeting mitochondrial dysfunction focuses on OBs, with limited studies on inhibiting activation of OCs. The integrated mechanism for bidirectionally regulating osteogenic-osteoclastic balance by ameliorating mitochondrial dysfunction remains unclear; ⑤ Molecular mechanisms underlying the synergistic effects of multi-metabolite CBD formulations are complex, requiring metabolomics and structural biology techniques to decipher their multi-target synergistic actions.

Given the above unresolved scientific issues and technical challenges in CBD targeting mitochondrial dysfunction for OP treatment, future research needs to focus on the following refined and actionable directions with clear technical means and core scientific problems to be solved: ① Establish an integrated and hierarchical “CBD-mitochondria-bone metabolism” research system. Taking bone cells (BMSCs, OBs, OCs) as the core research objects, combine multi-omics technologies including mitochondrial omics (mtDNA sequencing, mitochondrial proteomics), metabolomics (bone tissue/serum metabolic profiling), transcriptomics, and single-cell RNA sequencing (scRNA-seq) to decipher the spatiotemporal interaction networks among CBD active components, core regulators of mitochondrial dysfunction, and bone metabolism key signaling pathways; ② Strengthen in-depth research on the regulatory mechanism of mitochondrial dysfunction in OCs, which is relatively scarce in current studies. Taking OC mitochondrial biogenesis (PGC-1β/ERRα axis) and mitophagy (PINK1/Parkin/SIRT3 pathway) as the key entry points, combine *in vitro* cell models and *in vivo* animal models to explore the bidirectional regulatory pathways of CBD on bone remodeling; ③ Further advance mechanism-based translational medicine research, and design high-quality, multicenter, randomized, double-blind, placebo-controlled clinical trials targeting different osteoporosis subgroups. Based on the core indicators of mitochondrial dysfunction verified in basic research, set mitochondrial dysfunction-related molecular biomarkers and bone metabolism indicators as clinical efficacy evaluation endpoints, combined with bone mineral density and bone microstructure detection; ④ Advance the standardized research of CBD metabolites, including establishing quality control standards for CBD raw materials and optimizing extraction/purification processes of CBD effective parts. Integrate AI-assisted technologies, including network pharmacology, molecular docking, machine learning, and high-throughput screening, to screen CBD metabolites with high affinity and specificity for core targets of mitochondrial dysfunction, and carry out structural modification and optimization of metabolites; ⑤ Fully leverage the characteristics and advantages of network pharmacology combined with experimental validation (*in vitro* cell functional verification, *in vivo* animal efficacy evaluation) to construct a “CBD-mitochondrial dysfunction target-bone metabolism disease” network model. Based on the model, identify potential therapeutic metabolites from CBDs, classic formulations, and prescriptions, and clarify the synergistic regulatory effects of multiple metabolites in CBD on mitochondrial dysfunction.

In summary, targeting mitochondrial dysfunction with CBD represents a promising therapeutic strategy for OP. Elucidating the molecular mechanisms underlying mitochondrial dysfunction and establishing a comprehensive “CBD-mitochondria -bone metabolism” theoretical framework will provide novel targets and theoretical support for further developing CBD and exploring potential therapeutic strategies for OP. This approach holds promise as a potent weapon in the treatment of OP.

## Glossary

AMPK: adenylate-activated protein kinase
ATP: adenosine triphosphate
Bax: Bcl-2-associated X protein
BNIP3: Bcl-2 interacting protein 3
Bcl-2: B-cell lymphoma-2
β-FAO: β-oxidation
BMSC: bone marrow mesenchymal stem cell
BSP: bone-specific protein
Cyt C: cytochrome c
Drp1: dynamin-related protein 1
ERK: extracellular signal-regulated kinase
ERRα: estrogen-related receptor α
ETC: electron transport chain
FIS1: fission protein 1
FUNDC1: FUN14 domain-containing protein 1
GTP: guanosine triphosphate
IL-6: interleukin-6
IMM: inner mitochondrial membrane
LC3: microtubule-associated protein light chain-3
MAM: mitochondria-associated endoplasmic reticulum membrane
MDA: malondialdehyde
mtDNA: mitochondrial DNA
mTOR: mammalian target of rapamycin
mtROS: mitochondrial reactive oxygen species
NFATc1: nuclear factor of activated T-cells c1
NF-κB: nuclear factor kappa-B
NIX: NIP3-like protein X
Nrf1/2: nuclear respiratory factor 1/2
OB: osteoblast
OC: osteoclast
OCN: osteocalcin
OMM: outer mitochondrial membrane
OP: Osteoporosis
OPA1: fatty acid Optic atrophy 1
OPG: osteoprotegerin
OPN: osteopontin
OXPHOS: oxidative phosphorylation
PGC-1α: peroxisome proliferator-activated receptor gamma coactivator 1-alpha
PPAR: peroxisome proliferation-activated receptor
RANKL: receptor activator of NF-κB ligand
ROS: reactive oxygen species
Runx2: runt-related transcription factor 2
SIRT1/2: silent information regulator 1/2
TFAM: mitochondrial transcription factor A
TNF-α: tumor necrosis factor-alpha.
