# Supplementary material for: Mitigating mitochondrial dysfunction: a novel strategy for Chinese botanical drugs against osteoporosis
Source: Front Pharmacol. 2026 May 14;17:1809770. doi: 10.3389/fphar.2026.1809770 (PMC13216478; doi:10.3389/fphar.2026.1809770)
Supplement: Supplementary file 1 [file Supplementaryfile1.docx]

**Literature search strategy**

The research questions are as follows: (1) What are the key regulatory factors and pathways of mitochondrial dysfunction? (2) How does mitochondrial dysfunction influence the progression of osteoporosis? (3) How do Chinese botanical drugs alleviate osteoporosis by targeting mitochondrial dysfunction? The prospective protocol for this review was pre-established, with search terms and inclusion/exclusion criteria carefully selected to ensure comprehensive coverage of all relevant literature. A systematic literature search was performed on December 3, 2025, across the following electronic databases: Web of Science, PubMed, ScienceDirect, Embase and China National Knowledge Infrastructure (CNKI).

The search strategy employed free-text terms using the following keywords and Boolean operators:

(“Osteoporosis” OR “Osteoporosis, Age-Related” OR “Age-Related Osteoporosis” OR “Bone Loss, Age-Related” OR “Bone Losses, Age-Related” OR “Osteoporosis, Senile” “Osteoporoses, Senile” OR “Senile Osteoporoses” OR “Senile Osteoporosis”) AND (“Mitochondria” OR “Mitochondrion” OR “Mitochondrial Contraction” OR “Contraction, Mitochondrial” OR “Contractions, Mitochondrial” OR “Mitochondrial Contractions”) AND (“Chinese botanical drug” OR “Chinese traditional medicine” OR “medicine, Chinese traditional” OR “Chinese herbal medicine” OR “Chinese traditional medicine” OR “medicine, Chinese traditional” OR “Chinese Drugs, Plant” OR “Chinese Herbal Drugs” OR “Extracts, Chinese Plant”). External limitations were applied to each database, though specific constraints varied due to differences in search options.

No language restrictions were applied, but the search was primarily limited to studies published within the last 10 years to ensure currency of the findings. A limited number of older references of significant relevance were also included. The search strategies were adapted as necessary to accommodate the specific search interfaces and syntax requirements of each database.

**Inclusion and exclusion criteria**

Studies were included if they provided detailed mechanistic insights into the role of mitochondrial dysfunction in osteoporosis and the modulatory effects of Chinese botanical drugs. Eligible study types included original research articles, experimental studies, and clinical trials.

Exclusion criteria were as follows: (1) studies lacking sufficient evidence of mitochondrial dysfunction (n=3); (2) studies lacking sufficient evidence of Chinese botanical drug targeting mitochondrial dysfunction for the treatment of osteoporosis (n=41); (3) review articles, conference abstracts, editorials, and case reports (n=51).

**Literature screening and data extraction**

All retrieved records were imported into reference management software for deduplication. HL and SL independently screened the titles and abstracts of the remaining records against the eligibility criteria. Full texts of potentially relevant studies were then retrieved and assessed independently by the same two reviewers. Disagreements were resolved through discussion or consultation with a third reviewer.

Data were extracted from the included studies, focusing on the mechanisms of mitochondrial dysfunction regulation, the pathological role of mitochondrial dysfunction in osteoporosis, and the therapeutic effects and targets of Chinese botanical drugs. A detailed screening flowchart is presented in **Figure 1**.

**Identification of studies via database**

Records identified from Databases (n = 303)

**Identification**

Records after duplicates removed (n = 267)

Records excluded (n = 133)

Records screened (n = 170)

**Screening**

Full-text articles excluded, with reasons:

(1) studies lacking sufficient evidence of mitochondrial dysfunction (n=3);

(2) studies lacking sufficient evidence of Chinese botanical drugs targeting mitochondrial dysfunction for the treatment of osteoporosis (n=41);

(3) review articles, conference abstracts, editorials, and case reports (n=51).

Full-text articles assessed for eligibility (n = 39)

Articles included in review (n = 39)

**Included**

**Figure 1 Flowchart of the article selection process**

A total of 303 articles were initially identified from PubMed (n=15), Web of Science (n=20), Embase (n=70), ScienceDirect (n=119), and China National Knowledge Infrastructure (CNKI) (n=79). After removing duplicates, 267 records were screened. Following title and abstract screening, 170 full-text articles were assessed for eligibility. Exclusion criteria applied at the full-text stage included: (1) studies lacking sufficient evidence of mitochondrial dysfunction (n=3); (2) studies lacking sufficient evidence of Chinese botanical drugs targeting mitochondrial dysfunction for the treatment of osteoporosis (n=41); (3) review articles, conference abstracts, editorials, and case reports (n=51).Ultimately, 39 studies met the inclusion criteria and were included in the review.
